# Bergenin Monohydrate Attenuates Inflammatory Response via MAPK and NF-κB Pathways Against *Klebsiella pneumonia* Infection

**DOI:** 10.3389/fphar.2021.651664

**Published:** 2021-05-04

**Authors:** Qihe Tang, Qingyu Wang, Zhuojian Sun, Songyao Kang, Yimeng Fan, Zhihui Hao

**Affiliations:** ^1^ Agricultural Bio-pharmaceutical Laboratory, Qingdao Agricultural University, Qingdao, China; ^2^ College of Veterinary Medicine, Xinjiang Agricultural University, Urumqi, China; ^3^ National Centre for Veterinary Drug Safety Evaluation, College of Veterinary Medicine, China Agricultural University, Beijing, China

**Keywords:** *Klebsiella pneumoniae*, Bergenin monohydrate, anti-inflammatory, MAPK pathways, NF-κB pathways

## Abstract

**Background:** *Klebsiella pneumonia* has emerged as a critical pathogen causing severe clinical problems, such as pneumonia and sepsis. Meanwhile, intensified drug resistance induced by antibiotic therapy necessitates discovering novel and active molecules from Traditional Chinese Medicine (TCM) for treatment.

**Methods and results:** In this study, the isolated Bergenin monohydrate showed an anti-inflammatory effect in Klebsiella-infected mice. We initially investigated the anti-inflammatory effects and cytoprotection against oxidative stress *in vitro* and *in vivo*. Interestingly, a specific dose of Bm can effectively ameliorate lung injury and suppress the expression of inflammatory cytokines such as TNF-α, IL-6, IL-1β and PEG2. Moreover, Bm was also shown to reduced the levels of MPO, MDA and increased SOD and GSH activities. Moreover, we assessed the intracellular signaling molecules including p38, ERK, JNK, IκB, NF-κB-p65 by western blotting and verified through MAPK and NF-κB pathways inhibition experiments. These results reveal that Bm executed its effects via the classical MAPK signaling pathway and NF-κB pathway.

**Conclusion:** Given its underlying anti-inflammatory effect, Bm may be used as a promising therapeutic against Klebsiella-induced infection, thus providing a benefit for the future clinical therapy of pneumonia and medicine design.

## Introduction

*Klebsiella pneumoniae* (Kp), a nonmotile, facultatively anaerobic, gram-negative, rod-shaped bacterium, can cause acute lung injury in humans and is considered an urgent health concern (Peteranderl et al., 2017; Zhang et al., 2018). For adults in which Kp was the primary isolate, it caused a mortality rate of up to 50% in respiratory tract infections, with only 52% survival (Zhang et al., 2018; Choby et al., 2020). Currently, antibiotic administration remains a major strategy for treating Kp infections in the clinic (Bengoechea and Sa Pessoa, 2019). However, Kp has increasingly survived antibiotic treatment due to resistance or tolerance to the drugs. (Chew et al., 2017; Hossan et al., 2018; Chang et al., 2020). Hence, ample research space exists for the development of an efficient treatment to control Kp infection (Turan et al., 2018; Yang et al., 2018; Avgerinos et al., 2020).

Traditional Chinese medicine (TCM) has thousands of years of history in Asian countries. TCM studies have contributed to pharmacodynamic and pharmacokinetic development by generations of practitioners and researchers (Poivre and Duez, 2017; Chan and Ng, 2020). To date, several Chinese medicinal monomers extracted from traditional Chinese medicinal herbs have been widely used to treat inflammatory diseases of the lungs (Fan et al., 2018). For example, xanthohumol was reported to alleviate acute lung bacterial infection by adjusting the AMPK/GSK3β-Nrf2 signaling pathways (Lv et al., 2017a). At the same time, apigenin displayed protective effects in mice with pneumococcal pneumonia through pneumolysin cytolytic activity (Song et al., 2016). Likewise, daphnetin exhibited protective activity against t-BHP-induced oxidative injury and mitochondrial dysfunction by upregulating the Nrf2 antioxidant signaling pathway and activating JNK and ERK (Lv et al., 2017b). A previous study reported that Adenostemma Lavenia extract displayed a strong alleviating effect on pulmonary congestion, pneumonia, bacterial infections of the respiratory tract, edema, and inflammation (Batubara et al., 2020). In 2020, another study revealed that another extract of traditional Chinese medicine, baicalein, shows a significant effect against drug-resistant *S. aureus* infections by inhibiting the coagulase activity of vWbp and thus might be a promising therapeutic drug for bacterial pneumonia (Zhang et al., 2020). Notably, most bacteria rarely generate drug resistance to traditional Chinese medicine due to the unique and special properties of the therapeutic approach (Liao et al., 2018; Chan and Ng, 2020). This new perspective of investigating monomers from TCM has already contributed to modern medicine and has become a promising area of further research (Wang et al., 2018; Newman, 2020).

Over the last few years, our laboratory has set out to screen effective TCM molecules to treat Kp infection. Intriguingly, we found that Bergenin monohydrate (Bm) has multiple biological activities, suggesting its anti-inflammatory and immunomodulatory properties. Here, we confirmed that Bm exhibits a protective effect against Kp-induced cell damage and was effective in Kp-induced mice. In addition, we uncovered the involvement of the NF-κB and MAPK signaling pathways in the activity of Bm. This study might provide a potential reference for exploring therapeutic candidates derived from Chinese medicinal herbs to treat Kp infection.

## Materials and Methods

### Bacterial Strain, Chemicals and Animals

The Kp strain was purchased from the China Center of Industrial Culture Collection (CICC-10870). Bergenin monohydrate was obtained from Chengdu Herbpurify Co. Ltd., Sichuan, China, with a certified purity of ≥98. RAW 264.7 cells and WI-38 cells were obtained from our laboratory, and MEM and DMEM were provided by Procell Life Science and Technology Co. Ltd., Wuhan, China. The assay kits and instructions were purchased from Jiancheng Bioengineering Institute, Nanjing, China. The NF-κB pathway inhibitor (BAY 11-7082) and MAPK inhibitor (SB203580) were purchased from Sigma (Sigma-Aldrich). IκB, p-IκB, p68, p-p65, ERK1/2, p-ERK1/2, p65, p-p65, JNK, and p-JNK antibodies (rabbit antibodies) for western blot analysis were purchased from Beijing Boaosen Biotechnology Co., Ltd. Secondary antibodies (IgG-HRP) were obtained from Zhongshan Jinqiao Biological Technology Co., Ltd. Unless otherwise specified, the other materials were from Sigma-Aldrich (United States).

A total of 90 six-week-old BALB/c mice weighing 18–20 g were used in this study. All mice were fed ad libitum, kept in a pathogen-free environment, and maintained in groups of 5 per cage in a specific pathogen-free (SPF) animal room. All animal experiments, including any relevant details, were performed under the applicable guidelines and regulations of Experimental Animal Care and were approved by the Animal Welfare and Research Ethics Committee (Approval Number: SYXK (SD) 20170005).

### Determination of Bacterial Growth Curve and Preparation of Bacteria

The methods for bacterial growth and preparation were described in a previous study by our lab (Sun et al., 2019). Kp was strictly cultured in the microbial operation room following operating principles and cultured with graded Bm concentrations (0, 2.5, 5, 10 and 20 μg/ml) to the postexponential phase at 5% CO_2_, pH 7.0–7.4 and 37°C. Bacterial supernatant was collected for MTT, ROS. The rest of the Kp bacterial sample was utilized for cells experiments and animal experiment.

### MIC Determination Assay

Double-gradient dilution was applied to obtain a series of Bm solution concentrations, which were then sequentially added to the wells of a 96-well culture plate (Liao et al., 2018). The Kp broth was cultured to logarithmic growth phase and diluted to 1 × 10^7^ CFU/mL, and100 μL drops of the dilution were added to each well. Following incubation at 5% CO_2,_ pH 7.0–7.4, and 37°C for 24 h, we observed the turbidity of the broth. Transparency of the samples indicated that bacterial growth was inhibited at the corresponding concentration of the drug, and the lowest drug concentration that could inhibit bacterial growth was obtained. This concentration was considered the minimum inhibitory concentration (MIC).

### Cell Culture

After incubation in Dulbecco’s modified Eagle’s medium (DMEM) in 5% CO_2_ at 37°C, RAW 264.7 cells were cultured and adjusted to 2 × 104 cells/well, seeded into 96-well plates and incubated for 24 h. Bm was dissolved in DMEM. No significant treatment effect of Bm was found for Kp-infected RAW 264.7 cells in our experiments. Thus, RAW 264.7 cells were first administered Bm at different concentrations (final concentrations of 0, 2.5, 5, 10 and 20 μg/ml) and a positive control drug (dexamethasone, 100 μg/ml) for 1 h and then exposed to KP for 24 h.

### Measurement of ROS Production

Cultured RAW 264.7 cells were adjusted to 2 × 10^8^ cells/well and incubated in 6-well plates for 24 h. After that, the cells were treated with Bm at different concentrations (0, 2.5, 5, 10 and 20 μg/ml) in DMEM for 1 h. Then, the cells were infected with Kp for 24 h and washed twice with PBS. Subsequently, 50 μL DCFH-DA was added to the cells for 30 min without light. DCF fluorescence intensities were detected spectrophotometrically at excitation and emission wavelengths of 485 and 535 nm, respectively.

### Animal Experiment and Pathological Indexes

Ninety BALB/c mice were kept under SPF conditions and randomly divided into six groups:control group (saline only, 0.2 ml), Kp group (1 × 10^7^ CFU, 0.2 ml), Kp + Bm (75, 150 or 300 μg/kg, 0.2 ml) groups, Kp + DEX (100 μg/ml, 0.2 ml) group, Bm was diluted in saline. In the mouse model of Kp lung infection, Kp was administered intranasally, and the mice were treated with Bm via intraperitoneal injection 1 h before Kp infection. After the experiments, all the mice were sacrificed via carbon dioxide asphyxiation. Afterward, lung tissues were rinsed and fixed for further analysis, bronchoalveolar lavage fluid (BALF) was prepared for the following experiments. All vitro experiments were repeated at least three times, and all animal experiments were repeated twice for under identical conditions.

After euthanasia of mice by CO_2_ asphyxiation, the same lung tissues and BALF were collected as described in previous work (Liao et al., 2018). The levels of the representative inflammatory cytokines TNF-α, IL-6, IL-1β and PEG2 in BALF were then determined using specific ELISA kits.

Right lung tissues were excised from each mouse 48 h after Kp treatment. Then, we homogenized the lung tissues and dissolved them in extraction buffer for analysis of MPO, MDA, SOD and GSH contents. MPO and MDA contents were measured using assay kits to determine neutrophil accumulation and lipid peroxidation in lung tissues. BALF samples were centrifuged for 10 min at 4°C. The supernatant was then discarded, and cell pellets were resuspended in PBS. Finally, cells were stained using the Wright-Giemsa method, and the number of neutrophils, macrophages and total cells was determined. Lung tissue samples were homogenized in 10 ml normal saline. The homogenate was centrifuged at 2,500× g for 10 min, and the supernatant was collected. Counts of Kp organisms were obtained after plating serial dilutions of lung homogenates on Mueller-Hinton agar. After 24 h of incubation at 5% CO_2_ and 37°C, the colonies were counted and recorded.

### Histopathological Evaluation

Left lung tissues were excised 48 h after Kp infection, and histopathological examination was performed. Briefly, lung tissues from the mice were immersed in standard 10% neutral buffered formalin, dissected into 5 μm-thick sections, fixed, and then visualized via hematoxylin and eosin (H&E) staining. Images were acquired under a light microscope (Olympus, Japan). Comparison of the staining results was performed by research team members who were blinded to the grouping information.

### Western Blotting

Harvesting of protein lysates from RAW 264.7 cells and mouse lung tissues was performed according to the present study. Then, the same quantity of proteins (30 µg/lane) were subjected to electrophoresis in a 12% polyacrylamide gel, transferred to a PVDF membrane, and blocked with 5% FBS at room temperature for 1 h. The blots were then separately incubated with primary murine anti-human β-actin (1:5,000), IκB (1:1,000), phospho-IκB (1:1,000), p68 (1:1,000), phospho-p68 (1:1,000), p65 (1:1,000), phospho-p65 (1:1,000), ERK1/2 (1:1,000), and phospho-ERK1/2 (1:1,000) antibodies overnight at 4°C. β-Actin was used as an endogenous control and for normalization. The membranes were washed with TBST for 10 min and then incubated with anti-mouse IgG secondary antibody (1:100) for 30 min at room temperature. Finally, the membranes were visualized using an enhanced chemiluminescence (ECL) agent.

### Verification of Pathway Inhibition in WI 38 Cells

To verify that Bm attenuates the inflammatory response via the MAPK and NF-κB pathways in WI 38 cells, we cultured WI 38 cells in MEM containing 10% FBS, 1% P/S and 1% GlutaMax in an atmosphere of 95% air and 5% CO_2_. When the density of the WI 38 cells increased to 80%, we seeded them into 96-well plates at 1 × 10^5^ cells/well, incubated them for 24 h, and added SB203580 and BAY 11–7,082 at two different doses (10 and 20 μmol/L). After 1 h, 10 μg/ml Bm was added and incubated with the cells for another 1 h, and then, the plate was exposed to Kp for 24 h. The cytotoxic effects of these two pathway inhibitors were determined.

### Statistical Analysis

All the data were collected and analyzed using SPSS 22.0 software (IBM). The experimental groups were compared via one-way analysis of variance (ANOVA), and an LSD test was applied for multiple comparisons. In the western blotting analysis, densitometry analyses were quantified with ImageJ software. Statistical significance was defined as “*” for *p* < 0.05 and “**” for *p* < 0.01.

## Results

### Bergenin Monohydrate Rescues Cell Growth and ROS Production in *Klebsiella pneumonia*-Infected Cells

**FIGURE 1:**
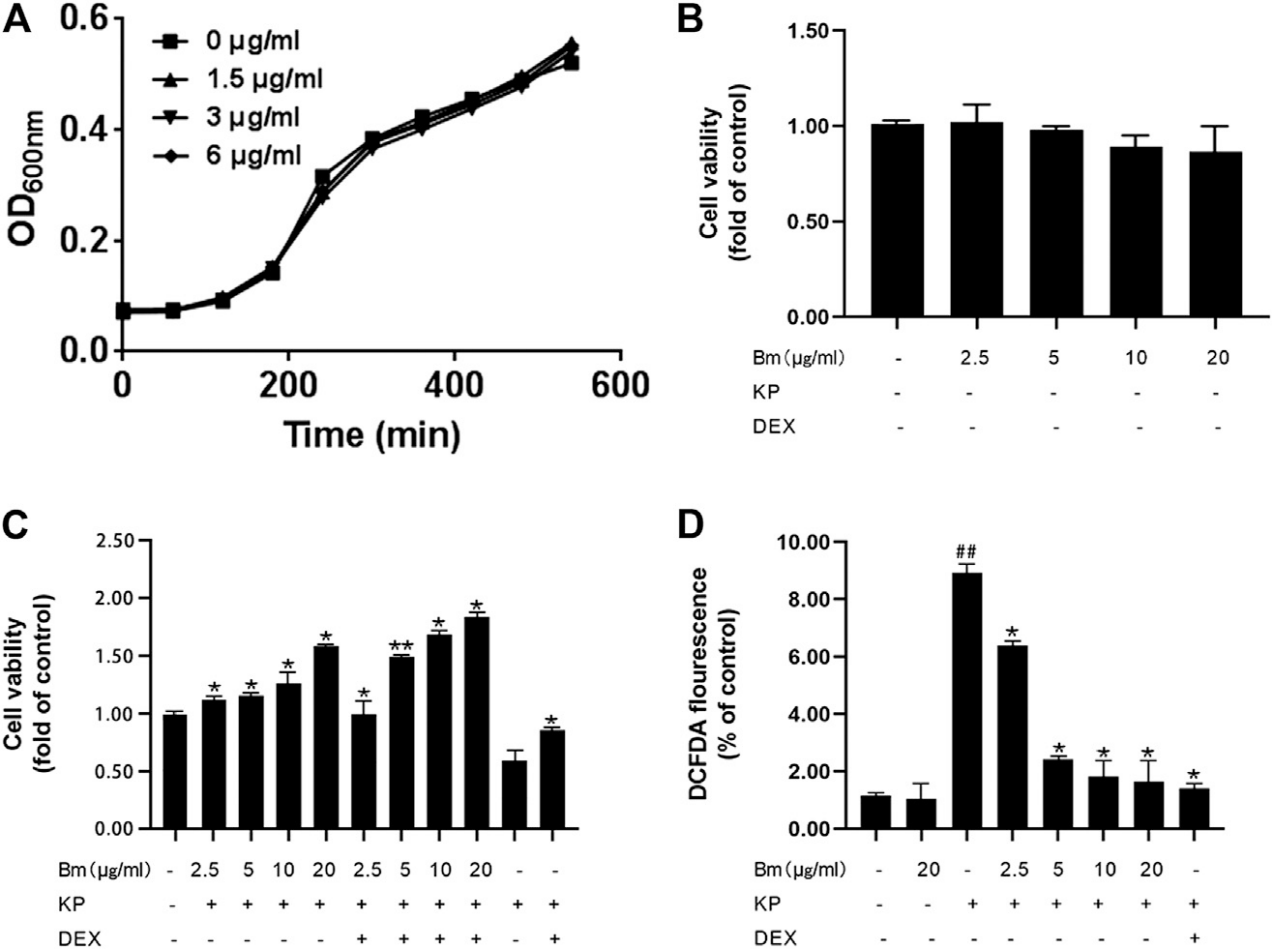
Effect of *Bergenin monohydrate* on *Klebsiella pneumonia*-infected RAW264.7 cell viability and ROS production **(A)** Viable cell count growth curves **(B)** Cell viability without *Klebsiella pneumonia* infection **(C)** Cell viability with both *Klebsiella pneumonia* infection and *Bergenin monohydrate* protection **(D)** ROS production with both *Klebsiella pneumonia* infection and *Bergenin monohydrate* protection. All data are presented as the means ± SEM (*n* = 5). ##*p*< 0.01 vs the Control group; *p < 0.05 and **p < 0.01 vs the *Klebsiella pneumonia* group.

The MIC of Bm was greater than 256 μg/ml, which indicated that Bm had no considerable effect on Kp growth (Supplementary Figure S1). In addition, growth curve assays (Figure 1A) showed no apparent difference between the groups, suggesting that Bm did not directly inhibit Kp growth. The results of MTT assays to measure cell viability indicated that 2.5, 5, 10 and 20 μg/ml Bm had no toxicity toward RAW 264.7 cells. (Figure 1B). Moreover, Kp significantly reduced cell viability, confirming successful modeling.

Furthermore, Bm protected RAW 264.7 cells from Kp infection (Figure 1C), and the protective efficacy increased with increasing Bm concentration. In addition, Bm enhanced the dexamethasone effect, which was most pronounced at 20 μg/ml. DCF fluorescence intensities were detected with a multidetection reader to calculate the percentage of DCF fluorescence in the control (Figure 1D). As indicated, the Kp-infected RAW 264.7 cells displayed higher ROS production than the Bm groups and the blank control group (p < 0.01). Notably, the ROS production in RAW 264.7 cells reached nearly the same level as that in the blank group at 20 μg/ml Bm, which was lower than that in the dexamethasone treatment group and even lower than that in the dexamethasone combined with Bm treatment group.

### Bergenin Monohydrate Treatment Significantly Decreased the *Klebsiella pneumonia* Content

**FIGURE 2:**
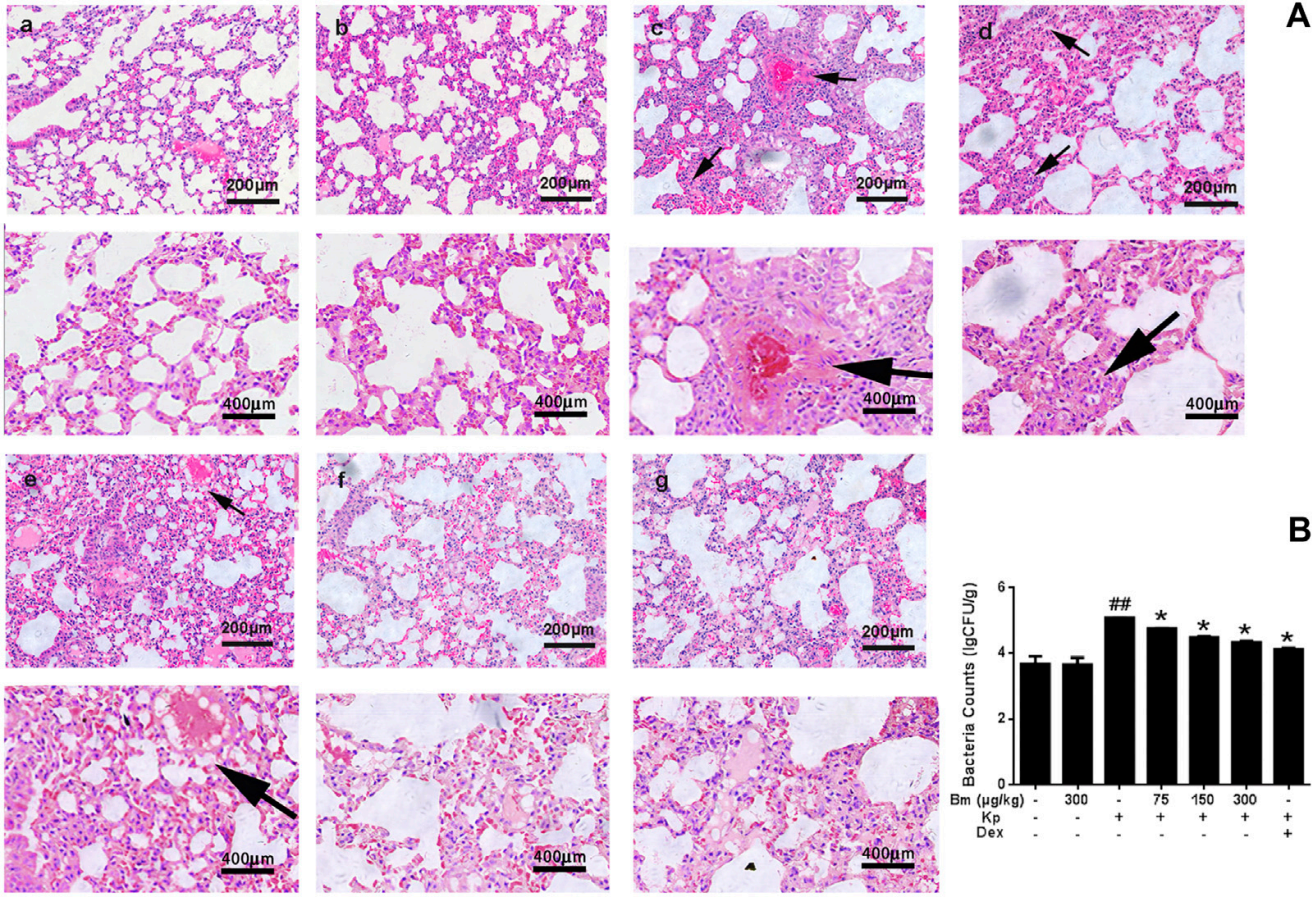
Characterization of lungs from the mouse model **(A)** Lung tissue structure was observed with H&E staining: a) blank control group, b) *Bergenin monohydrate* treatment group (300 μg/kg), c) *Klebsiella pneumonia*-infected group, e-f) *Klebsiella pneumonia*-infected mice treated with *Bergenin monohydrate* (75, 150, 300 μg/kg), g) Dex group (black arrows: pathological changes) **(B)** Lung bacterial content changes after *Klebsiella pneumonia* infection. ##*p* < 0.01 vs the Control group; *p < 0.05 and **p < 0.01 vs the *Klebsiella pneumonia* group; $*p* < 0.05 vs the Dex group.

Histological changes in lung tissues were assessed by H&E staining (Figure 2A). There were no signs of structural damage in the lung tissue after 300 μg/kg Bm treatment (Figures 2A,b). The Kp group (Figures 2A,c) had the thickest alveolar walls, red blood cells were found in the alveolar cavity, and the capillary walls were distinctly expanded. Compared with the Kp group, the Bm-treated groups (Figures 2A,e,f) showed effectively alleviated lung tissue injury, including less accumulation of inflammatory cells, thinner alveolar walls and reduced alveolar hemorrhage. All changes occurred in a dose-dependent manner. In particular, the lung tissue status in the high-dose Bm treatment group (Figures 2A,f) was similar to that in the dexamethasone group (Figures 2A,g) and tended to be close to that in the blank group. Edema status was also evaluated by examining pulmonary bacterial content in lung tissues. As shown in Figure 2B, the Kp content in the Kp group was substantially higher than in the other groups, while groups pretreated with Bm at 75, 150, and 300 μg/kg showed reduced Kp content in the lungs by 4.76, 4.48, and 4.32 L g CFU/g, respectively. These findings indicate that Bm had significant effects in lowering the amount of Kp bacteria within the lungs.

ELISA measurement of TNF-α, IL-6, IL-1β and PEG2 contents after Kp treatment for 48 h.

**FIGURE 3:**
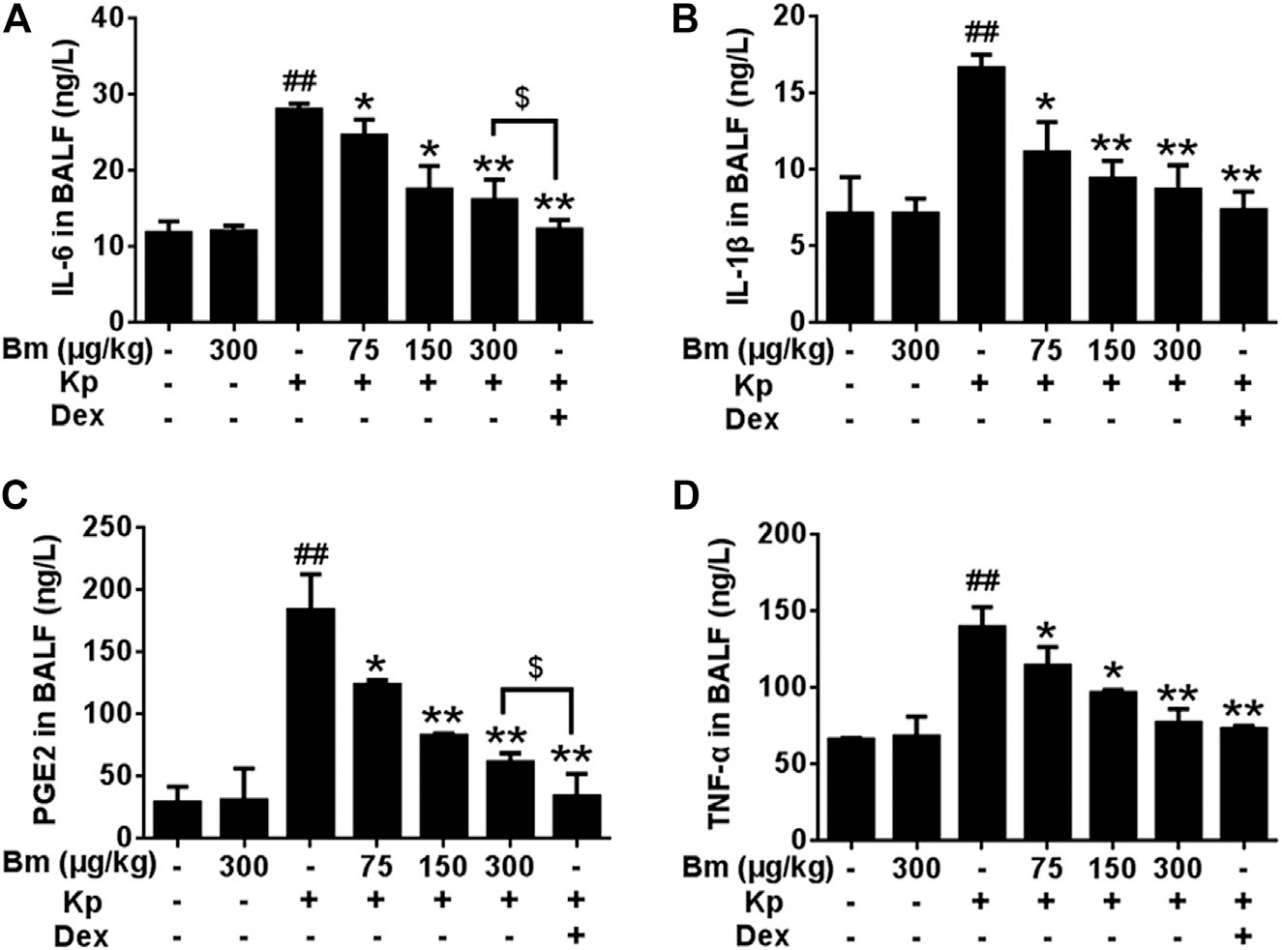
Effects of *Bergenin monohydrate* on IL-1β **(A)**, IL-6 **(B)**, PEG2 **(C)** and TNF-α **(D)** production and secretion in BALF from *Klebsiella pneumonia*-induced pneumonia mice. All data are presented as the means ± SEM (*n* = 5). ##*p* < 0.01 vs the Control group; *p < 0.05 and **p < 0.01 vs the *Klebsiella pneumonia* group; $*p* < 0.05 vs the Dex group.

As indicated in Figures 3A-D, TNF-α, IL-6, IL-1β and PEG2 contents in BALF were significantly increased after Kp administration compared with the control group. After treatment with Bm (75, 150, and 300 μg/kg), TNF-α, IL-6, IL-1β, and PEG2 levels in Kp-induced pneumonia mice exhibited a downward trend in a dose-dependent manner.

### Effects of Bergenin Monohydrate on Cell Counts in *Klebsiella pneumonia*-Infected Mice

**FIGURE 4:**
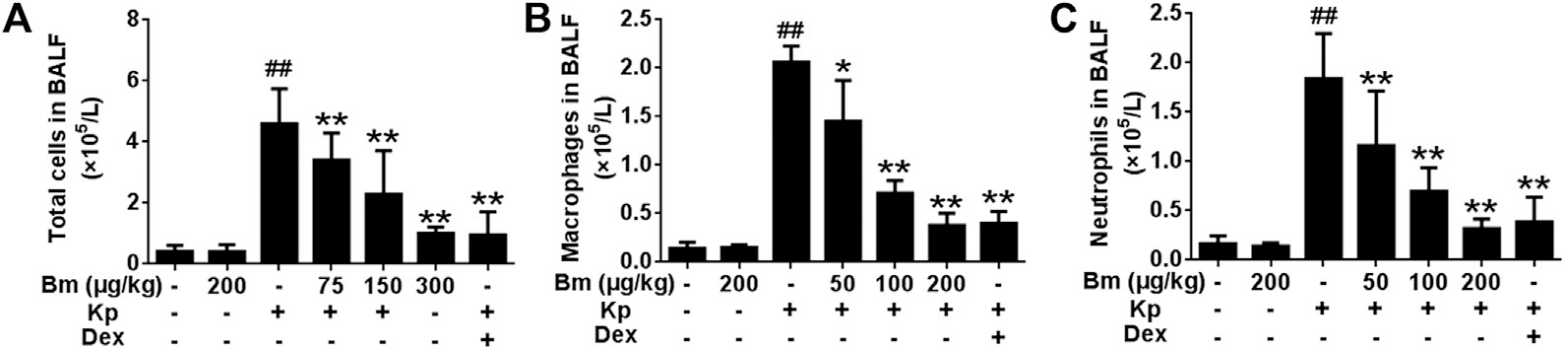
Effects of *Bergenin monohydrate* on the number of total cells, neutrophils and macrophages after *Klebsiella pneumonia* infection **(A)** Statistics of total cells in BALF **(B)** Statistics of macrophages in BALF **(C)** Statistics of neutrophils in BALF. All data are presented as the means ± SEM (*n* = 5). ##*p* < 0.01 vs the Control group; *p < 0.05 and **p < 0.01 vs the *Klebsiella pneumonia* group.

As illustrated in Figures 4A-C, Kp-infected mice showed increased total cells, macrophages, and neutrophils compared with the control group. Strikingly, after treatment with Bm at three doses (75, 150, and 300 μg/kg), cell numbers were effectively reduced in the mice.

Bm treatment restored the MPO, MDA, SOD and GSH levels in Kp-infected mice.

**FIGURE 5:**
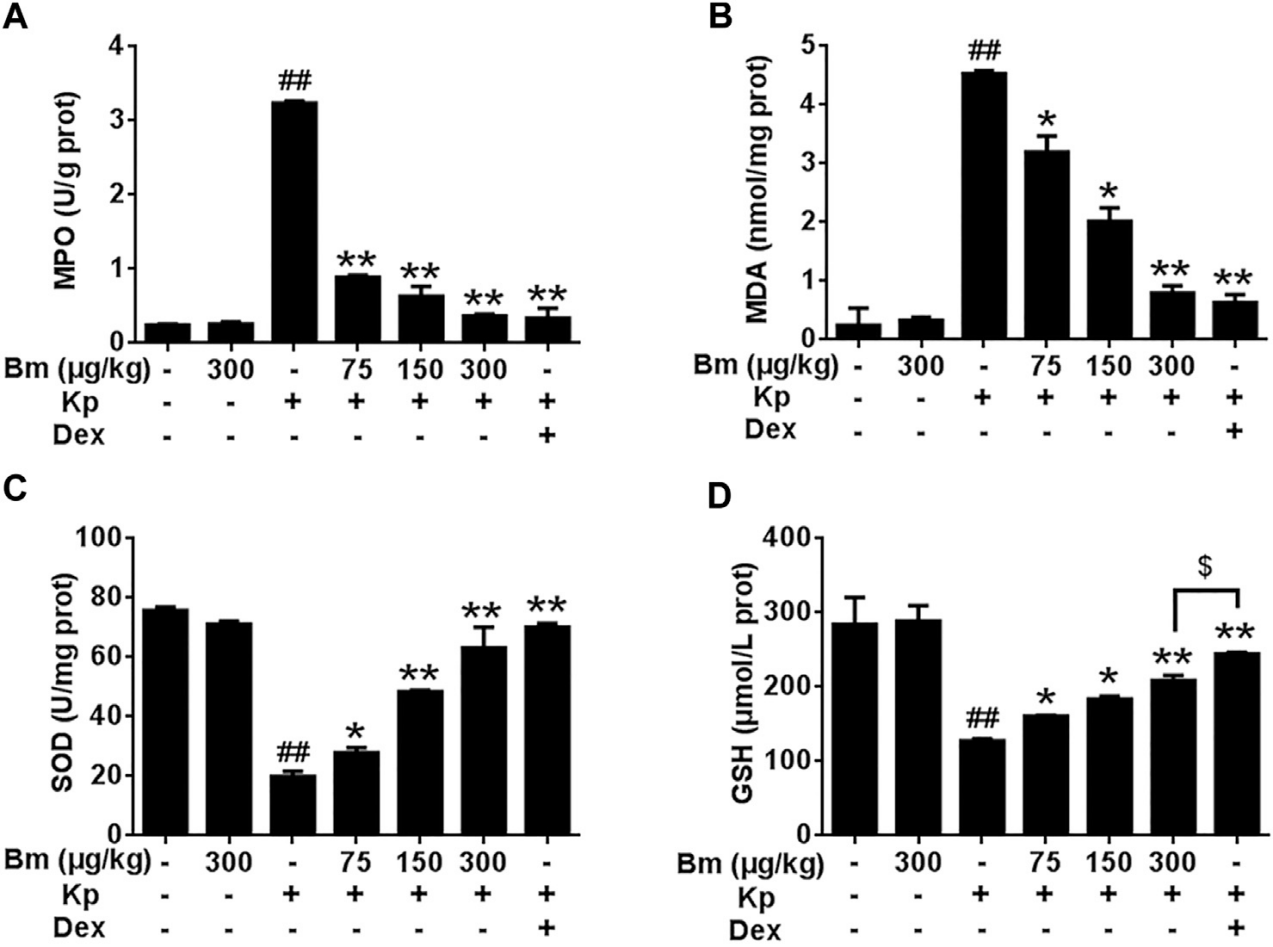
Effects of *Bergenin monohydrate* on MPO **(A)**, MDA **(B)**, GSH **(C)** and SOD **(D)** levels in lung tissues after *Klebsiella pneumonia* infection. *p < 0.05 and **p < 0.01 indicate significant differences compared with the *Klebsiella pneumonia* only group. All data are presented as the means ± SEM (*n* = 5 in each group). ##*p* < 0.01 vs the Control group; *p < 0.05 and **p < 0.01 vs the *Klebsiella pneumonia* group; $*p* < 0.05 vs the Dex group.

As shown in Figures 5A-D, Kp significantly elevated MPO and MDA levels compared with levels in the other groups. Groups pretreated with Bm at 75, 150, and 300 μg/kg showed decreased MPO levels by 4.07, 3.07, and 1.52 U/g, respectively, and decreased MDA levels by 3.20, 2.02, and 0.80 nmol/mg, respectively. Meanwhile, Kp dramatically reduced the levels of SOD and GSH. Groups pretreated with Bm exhibited increased SOD levels by 31.22, 45.40, and 52.65 U/mg, respectively, and increased GSH levels by 160.46, 183.46, and 208.41 μmol/L, respectively.

### Bergenin Monohydrate Inhibited Kp-Induced NF-κB and MAPK Signaling Pathway Activation

**FIGURE 6:**
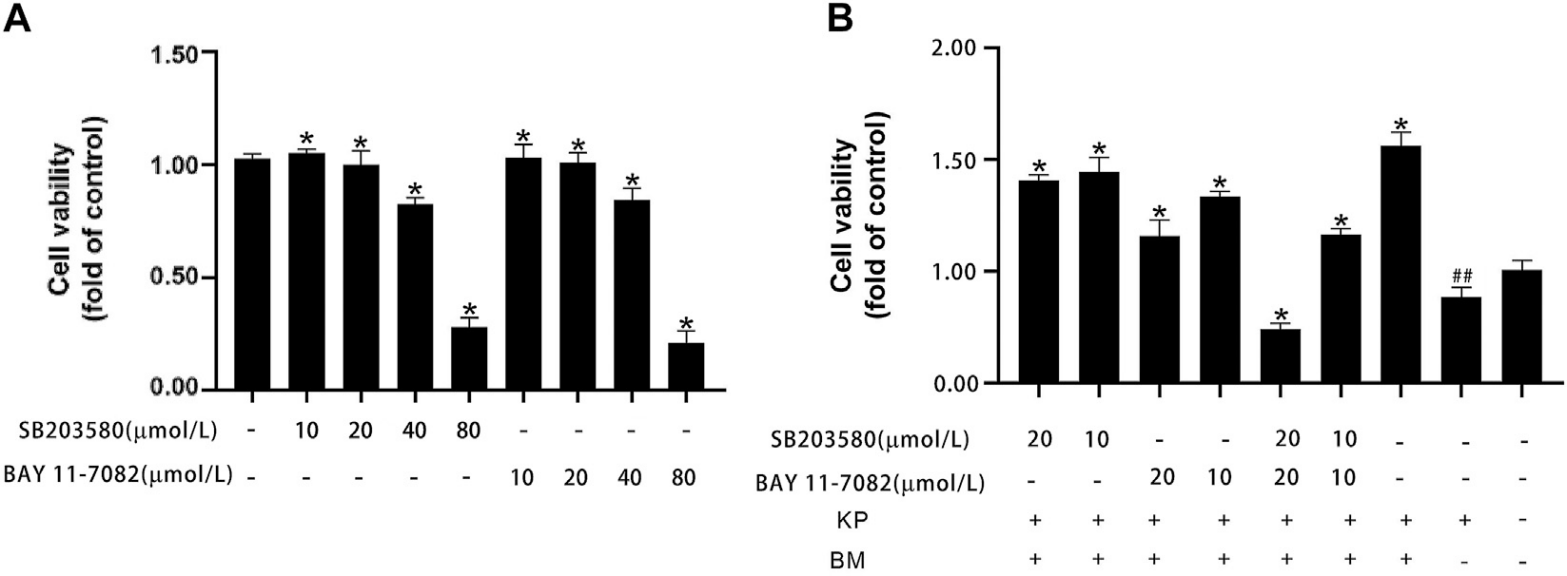
Effects of *Bergenin monohydrate* on the MAPK and NF-κB signaling pathways **(A)** MAPK and NF-κB pathway inhibitors were cytotoxic to WI 38 cells at higher concentrations **(B)** Cell viability of *Bergenin monohydrate-*treated and *Klebsiella pneumonia*-infected WI 38 cells after treatment with different doses of MAPK and NF-κB pathway inhibitors. ##*p* < 0.01 vs the Control group; *p < 0.05 and **p < 0.01 vs the *Klebsiella pneumonia* group.

We first examined the effect of a MAPK pathway inhibitor (SB203580) and NF-κB pathway inhibitor (BAY 11-7082) on WI38 cell viability. SB203580 and BAY 11-7082 at concentrations up to 20 µM barely altered cell viability, but at concentrations above 20 μM, they were cytotoxic (Figure 6A). Within safety thresholds, both the MAPK pathway inhibitor and NF-κB pathway inhibitor significantly reduced the protective effect of Bm against Kp infection in WI38 cells. The inhibitory effect of high concentrations was more robust than that of low concentrations (Figure 6B). Among them, the cell survival rate was even lower than that in the blank control group when 20 µM MAPK pathway inhibitor and NF-κB pathway inhibitor were added. However, without inhibitor interference, the cell survival rate was the highest, and Bm showed a relatively noticeable therapeutic effect.

**FIGURE 7:**
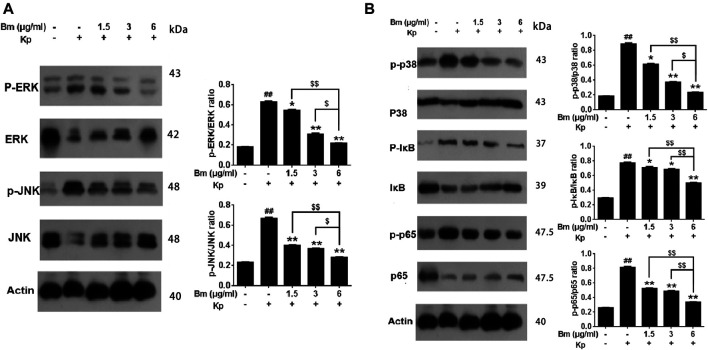
Effects of *Bergenin monohydrate* on the NF-κB and MAPK pathways in *Klebsiella pneumonia*-infected RAW264.7 cells determined by western blotting. Effects of *Bergenin monohydrate* on the expression of ERK, P38 and JNK in the MAPK pathway in RAW264.7 cells. Effects of *Bergenin monohydrate* on the expression of IκB/P65 in the NF-κB pathway in RAW264.7 cells **(A)** Effects on MAPK pathways in *Klebsiella pneumonia-infected* RAW264.7 cells **(B)** Effects on NF-κB pathways in *Klebsiella pneumonia-infected* RAW264.7 cells. All data are presented as the means ± SEM (*n* = 5 in each group). ##*p* < 0.01 vs the Control group; *p < 0.05 and **p < 0.01 vs the *Klebsiella pneumonia* group; $*p* < 0.05 and $$*p* < 0.01 vs the high-dose group.

**FIGURE 8:**
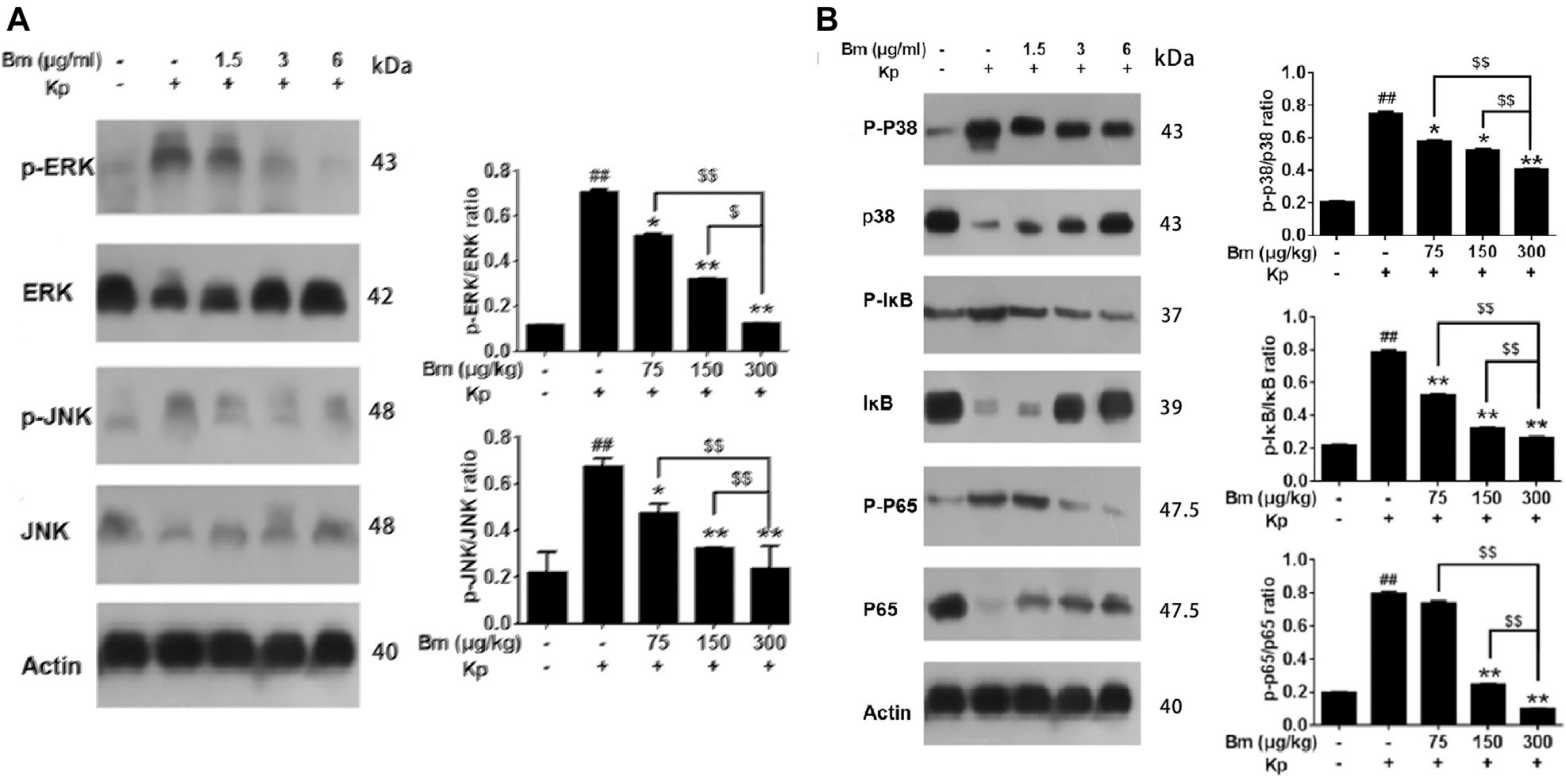
Effects of *Bergenin monohydrate* (Bm) on the NF-κB and MAPK pathways in *Klebsiella pneumonia*-infected mice determined by western blotting. Effects of B*ergenin monohydrate* on the expression of ERK, P38 and JNK in the MAPK pathway in *Klebsiella pneumonia*-infected mice. Effects of *Bergenin monohydrate* on the expression of IκB and P65 in the NF-κB pathway in *Klebsiella pneumonia*-infected mice **(A)** Effects on MAPK pathways in *Klebsiella pneumonia*-infected mice **(B)** Effects on NF-κB pathways in *Klebsiella pneumonia*-infected mice. All data are presented as the means ± SEM (*n* = 5 in each group). ##*p* < 0.01 vs the Control group; *p < 0.05 and **p < 0.01 vs the *Klebsiella pneumonia* group; $*p* < 0.05 and $$*p* < 0.01 vs the high-dose group.

As shown in the western blotting results, compared with the control and Bm groups after infection with Kp, the Kp group displayed higher levels of p38, ERK, and JNK phosphorylation (Figures 7A,B). There was an obvious dose-dependent effect among the groups in which pretreatment with Bm drastically suppressed phosphorylation of the three MAPK subfamilies p38, ERK, and JNK. Likewise, we examined IκB and NF-κB p65 subunits. We found that the Kp group displayed higher levels of IκB and NF-κB p65 phosphorylation than the other groups. In addition, *in vivo* studies confirmed a similar trend in p38, ERK, and JNK phosphorylation as observed in the *in vitro* experiments (Figures 8A,B). Hence, Bm efficiently inhibited the MAPK and NF-κB signaling pathways while attenuating the inflammatory response in Kp mice.

## Discussion

*Klebsiella pneumonia* is a crucial type of bacteria in the genus *Klebsiella* and accounts for more than 95% of *Klebsiella* infections (Raetz et al., 1991; Geetha et al., 2020; Ota et al., 2020). Kp infection is acknowledged to be severe and challenging to treat, causing a high mortality rate. In the past several years, antibiotics have been used to clear up this pneumonia, posing significant challenges due to multidrug resistance (Bengoechea and Sa Pessoa, 2019). Increasing antibiotic resistance necessitates the development of new drugs produced from bioactive natural products owing to their broad-spectrum pharmacological activity and low toxicity (Mohr, 2016; Pilehvar et al., 2016; Dodds, 2017). TCM monomers and their derivatives are believed to be very helpful in resolution of inflammatory responses, and their bioactive function and mechanism have been explored in recent years. As a representative TCM monomer, Bergenia purpurascens Qi Q., et al., 2018 which is a potent compound extracted from Bergenia purpurascens, has been demonstrated to exert numerous pharmacological effects, including antibacterial, analgesic, anti-inflammatory and antitumor activities (Fisher et al., 2017; Wu et al., 2017).

In the present study, both *in vitro* and *in vivo* experiments showed that Bm can suppress the expression of major inflammatory cytokines in Kp-infected cells and ameliorate lung injury in Kp-infected mice. ROS measurements in RAW 264.7 cells revealed a dose-dependent protective effect of Bm. Histological changes and pulmonary bacterial count results indicated that Bm reduced Kp bacteria levels while improving lung tissue histopathology. Bm is the main medicinal component of *Ardisia japonica*, and a large number of studies have shown that *Ardisia japonica* can be employed clinically to treat lung injury diseases, such as tuberculosis (Wang et al., 2016; Choi et al.,2004). In addition, Bm can prevent damage to the spleen and thymus by enhancing cellular immunity and cellular antioxidative activity (Qi et al., 2018) and promote anti-inflammatory and immunoregulatory effects. These results are consistent with our cell and animal experiment results. In the initial inflammatory process induced by Kp, lung tissue damage induces neutrophil infiltration, and one significant index of neutrophil infiltration is MPO Lv et al., 2017 MDA is produced by cell membranes and can be used to reflect free radical metabolism and the state of cells attacked by free radicals (Reichmann et al., 2018). GSH and SOD are key indicators of oxidative stress, and their ratio reflects the redox state of the cellular environment and, consequently, is a cellular health measure (Petrov et al., 2016). In our study, treatment with Bm caused a dose-dependent decrease in MPO and MDA and an increase in SOD and GSH levels, thereby protecting cells against oxidative insult by Kp. As a side observation, the therapeutic effect of Bm was consistent with the pathway inhibition experiment results in human fibroblasts (WI38 cells), heralding the possibility of effective therapy for human *Klebsiella pneumonia*.

TNF-α, IL-6, IL-1β and PEG2 proinflammatory cytokines, including TNF, IL-1, IL-6 and IL-8, are small molecular peptides that can regulate a variety of physiological functions and increase the expression of proinflammatory cytokines (Fann et al., 2018). These important cytokines are interrelated and involved in regulating the inflammatory response (Yang et al., 2020). IL-6 and IL-1β are involved in the inflammatory response in organs and tissues of mammals and are generally known as triggers of pathologic pain (Hudock et al., 2020). In 2020, IL-1β, IL-6, and other proteins were found to be correlated with pulmonary inflammation with extensive lung involvement, as seen in COVID-19 patients. TNF-α improves the levels of PEG2 and bradykinin, which are also thought to influence the TNF-α/TNF receptor signaling pathway and promote the development of pulmonary endothelial cell injury (Nishida et al., 2020). In 2018, a study reported that knockdown of TNF-α alleviated the severity of lung injury; thus, TNF-α might be a key target in injury and repair related to the lung (Qi et al., 2018). In the present study, the ELISA results indicated that TNF-α, IL-6, IL-1β and PEG2 levels in Kp-induced pneumonia mice were downregulated during Bm treatment, suggesting that Bm attenuates intestinal inflammation by decreasing the levels of these cytokines in lung tissues and cells.

To explore further pathway mechanisms, we investigated whether Bm regulates the MAPK and NF-κB signaling pathways to execute protective and anti-inflammatory actions. Western blotting and ELISA results revealed that the levels of the proinflammatory cytokines TNF-α, IL-6, IL-1β and PEG2; the MAPK subfamilies p38, ERK, and JNK; and the NF-κB subfamilies IκB and NF-κB-p65 exhibited a downward trend in a dose-dependent manner with Bm treatment after Kp infection. Both the MAPK and NF-κB signaling pathways are regarded as classical and significant anti-inflammatory pathways (Sun, 2017; Kim et al., 2019; Ali et al., 2020; Ren et al., 2020). The pathway inhibition experiments in the present study confirmed the involvement of the MAPK signaling pathway and NF-kappaB signaling pathway in the activity of Bm against Kp, and good agreement among pathway inhibition experiments and ELISA and western blotting results was found. MAPKs are a family of serine/threonine protein kinases that mediate fundamental biological processes, signal transduction and cell responses and phosphorylate various substrate proteins, including proinflammatory transcription factors; thus, MAPKs are potential targets for anti-inflammatory therapeutics (Liang et al., 2014). In mammals, The MAPK family includes the extracellular signal-regulated kinase (ERK), Jun N-terminal kinase (JNK) and p38 pathways (Zhao et al., 2020). Together with the findings obtained in this study, we found that after Kp infection, Bm could regulate the MAPK pathway by inactivating JNK, ERK and p38. NF-κB is another prominent transcription factor involved in various functions affecting cellular behavior (Victor et al., 2005) and is considered the central mediator of the inflammatory response and a leading participant in normal immune processes. NF-κB is activated by the MAPK pathway to regulate antimicrobial and inflammatory responses. As one of the downstream ERK/MAPK signaling pathway pathways, NF-kB plays an important role in inflammatory processes (Baldwin, 1996; Urbano et al., 2014; Soleimani et al., 2019). In the nucleus, activated NF-kB is phosphorylated as a transcription factor of inflammatory genes (Kim et al., 2019; Mishra et al., 2020; Soleimani et al., 2020). IκB is a negative regulator of the NF-κB pathway, and activation of IκB promotes NF-κB p65 translocation to the cell nucleus. (Qi et al., 2018; Xu et al., 2019). Moreover, several previous studies have illustrated that activated NF-κB regulates the expression of genes such as IL-1β, IL-6, and TNF-α and promotes the occurrence, development and pathogenesis of autoimmune diseases, chronic infections and cancer (Nold-Petry et al., 2015; Stassi et al., 2017; Wilson et al., 2020). The results of this study are consistent with those of the above studies, and similarly, our study provides an experimental basis for application of Bm, potentially leading to discovery of other effects and side effects.

The study had a few limitations. First of all, Bm has not been found an obvious inhibition effect for Kp growth and protective effect for Kp-infected Raw cells, but in animal experiments, it demonstrated a good anti-inflammatory effect. This phenomenon attracted our attention. By reviewing the available literature, we have found that conditions *in vitro* are unable to mimic that *in vivo* environment with hypoxia, hypoglycemia, and other metabolic changes (Su et al., 2019), although some compound didn’t show an inhibitory effect on pathogens *in vitro*, they can actually inhibit the growth of pathogens by changing the microenvironment *in vivo* (Liu et al., 2017). However, the specific mechanisms require further investigation. Moreover, the influence of Bm for MAPK and NF-κB signaling pathways were confirmed, but the mechanisms underlying the effects of Bm in other signaling pathways are not completely understood and require further investigation.

In conclusion, our results demonstrated a dose-dependent protective role of Bm in Kp-infected cells and mice through modulation of inflammatory cytokine expression. Bm exerted anti-inflammatory action by regulating Iκ-Bα and p65 phosphorylation and regulated the MAPK signaling pathway by inhibiting p38, ERK, and JNK phosphorylation after Kp infection. Thus, our findings provide evidence that Bm may be a promising therapeutic agent for Kp prophylaxis.

## Data Availability

The raw data supporting the conclusions of this article will be made available by the authors, without undue reservation.
