# Supplementary material for: Bergenin Monohydrate Attenuates Inflammatory Response via MAPK and NF-κB Pathways Against Klebsiella pneumonia Infection
Source: Front Pharmacol. 2021 May 4;12:651664. doi: 10.3389/fphar.2021.651664 (PMC8129520; doi:10.3389/fphar.2021.651664)
Supplement: Supplementary file 1 [file DataSheet1.pdf]

Supplementary Material

Bergenin monohydrate attenuates inflammatory response via MAPK and NF-κB pathways against Klebsiella pneumonia Infection

Qihe Tang[2,3]†, Qingyu Wang[1]†, Zhuojian Sun[2], Songyao Kang[2], Yimeng Fan[1],Zhihui Hao[1*]

† Qihe Tang[2,3] and Qingyu Wang[1] contributed equally to this article.

[1] National Centre for Veterinary Drug Safety Evaluation, College of Veterinary Medicine, China Agricultural University, Beijing, China

[2] Agricultural Bio-pharmaceutical Laboratory, Qingdao Agricultural University, Qingdao, China.

[3] College of Veterinary Medicine, Xinjiang Agricultural University, Urumqi, China

*** Correspondence:**

Zhihui Hao. National Centre for Veterinary Drug Safety Evaluation, College of Veterinary Medicine, China Agricultural University, Beijing,100086, China

E-mail: haozhihui@cau.edu.cn

1 Supplementary Figures

1. 1. The results of MIC determination

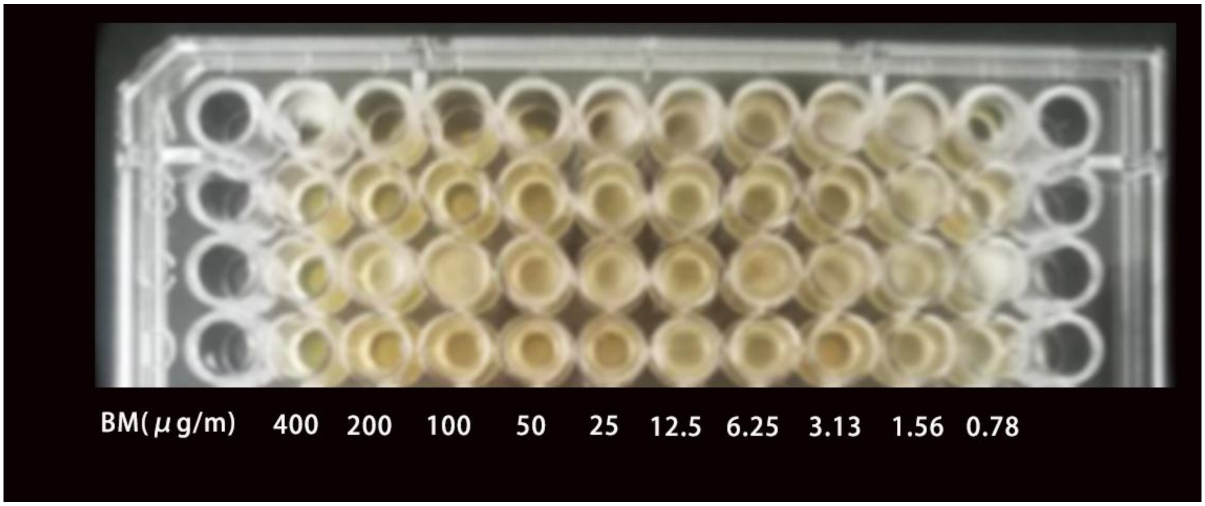

Figure 1 MICs of different concentration of *Bergenin monohydrate* (400, 200, 100, 50, 25, 12.5, 6.25, 3.13, 1.56, 0.78 μg/m) against *Klebsiella pneumonia*

1.2 Figures of Western blotting experiment results in full length.

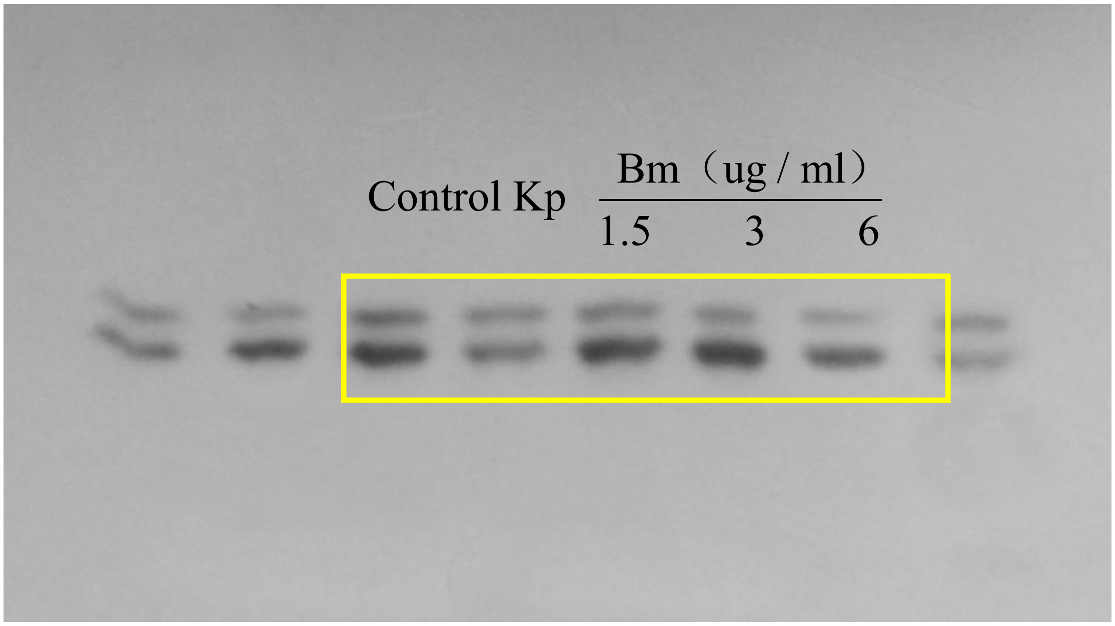

22593 30604 38723 11829 41180 35818 20468 14358

Figure 7. p-ERK

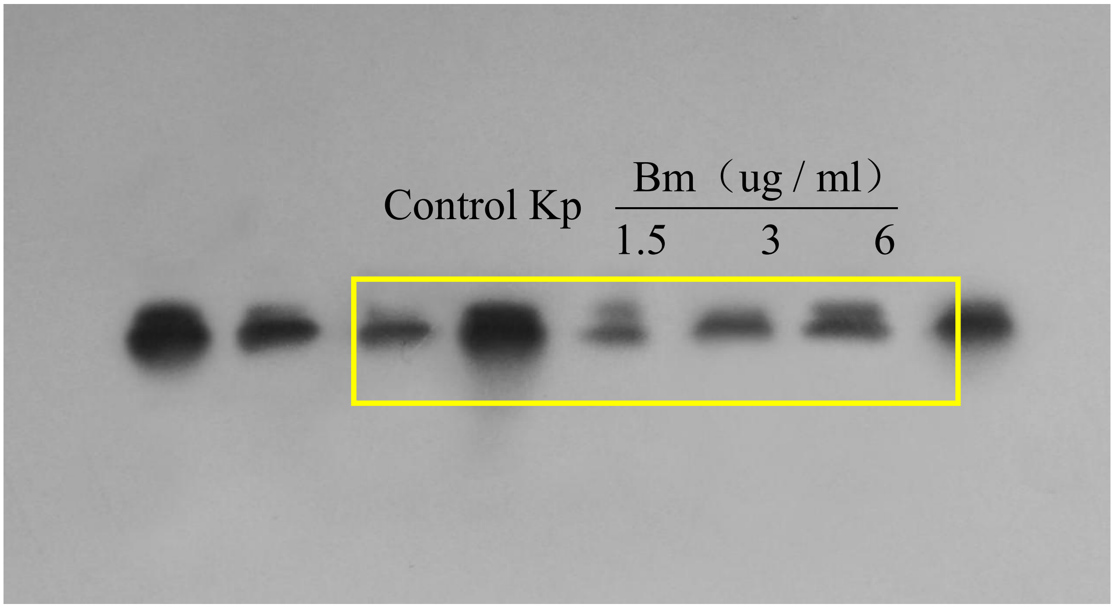

43717 30556 25291 49595 14149 16738 19531 25735

Figure 7. ERK

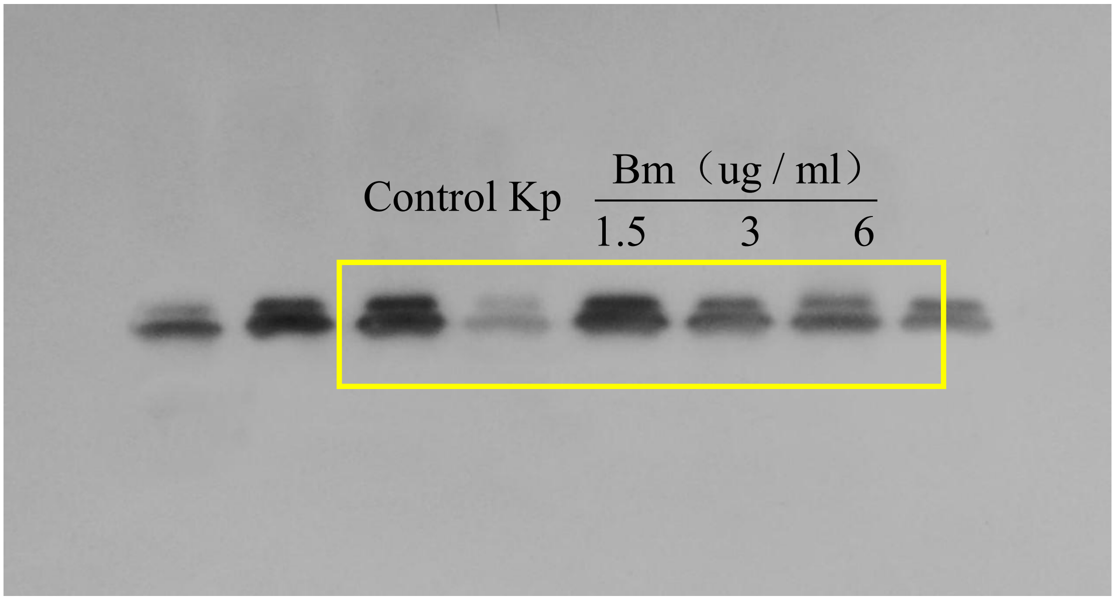

28324 38381 40520 15264 43791 26296 24062 18542

Figure 7. p-JNK

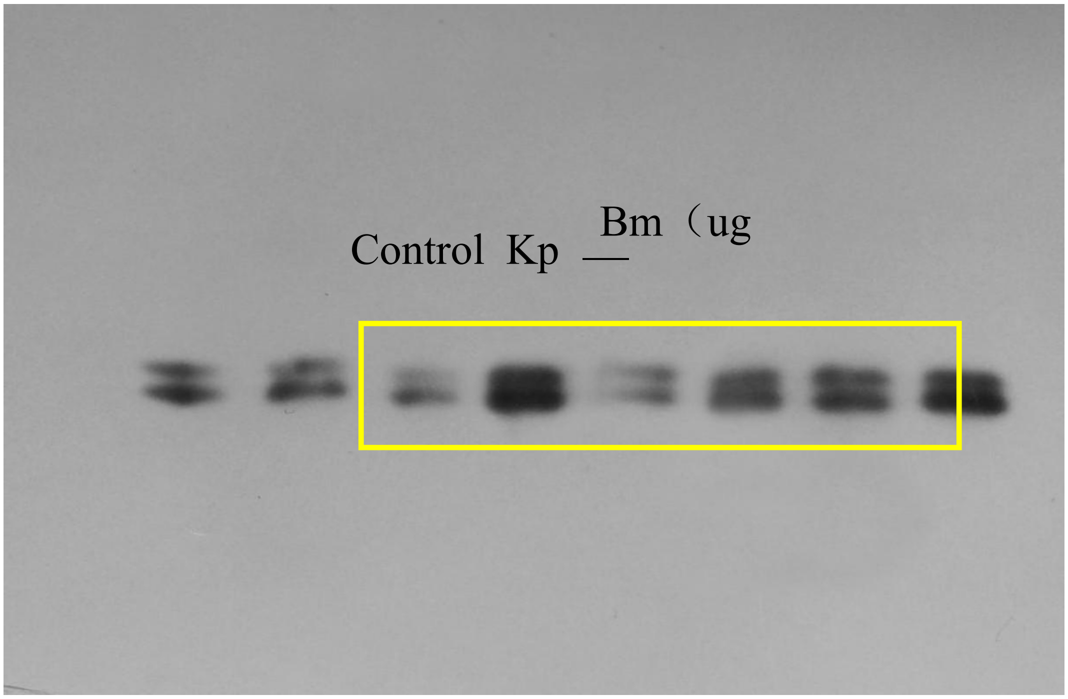

31684 28743 20875 55214 19530 29147 36152 43959

Figure 7. JNK

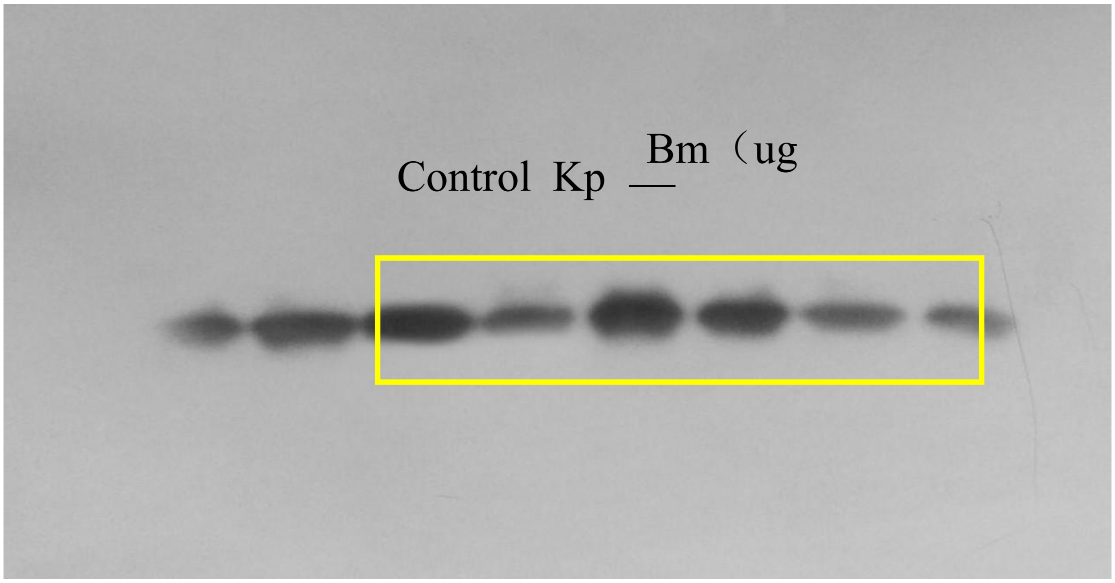

27765 48267 52327 12059 58269 40198 24585 15549

Figure 7. p-p38

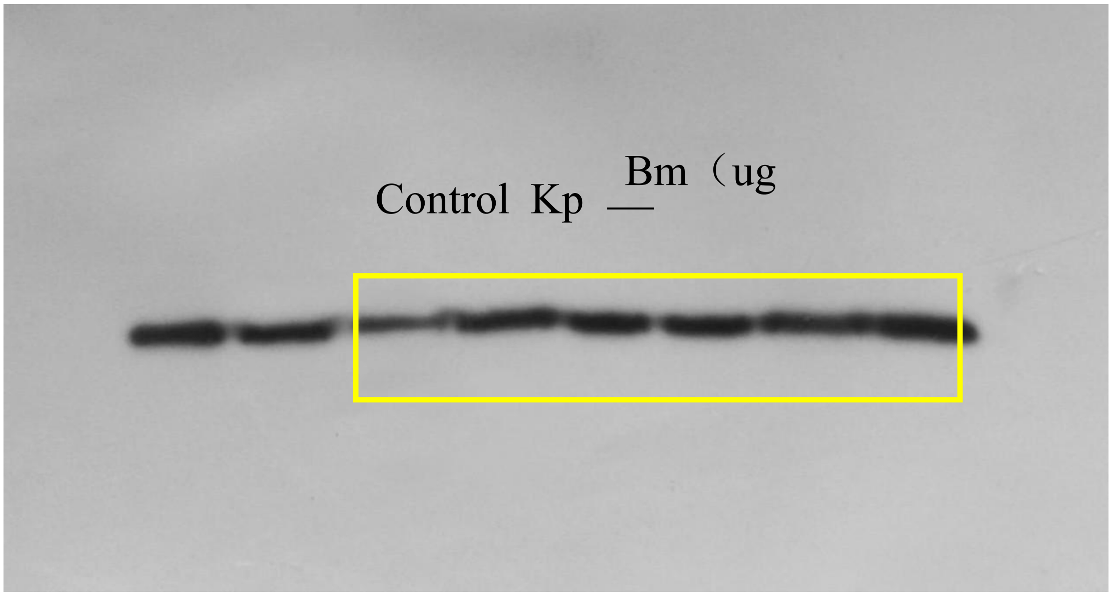

55627 48692 24600 58001 20599 45320 53605 56925

Figure 7. P38

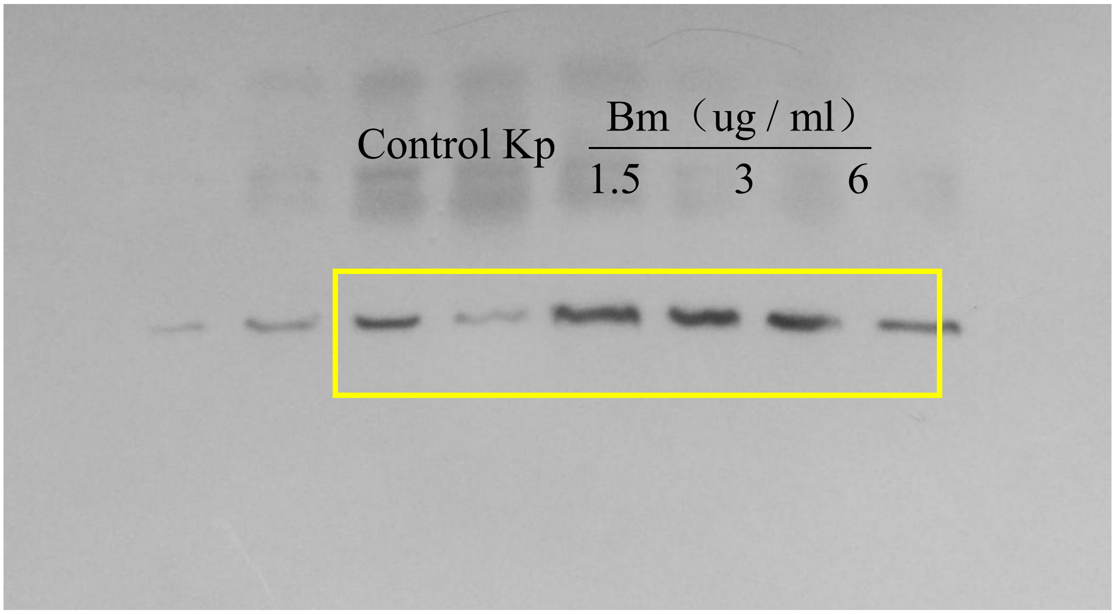

4639 15459 21309 19285 50767 46620 44980 32686

Figure 7. P-IkB

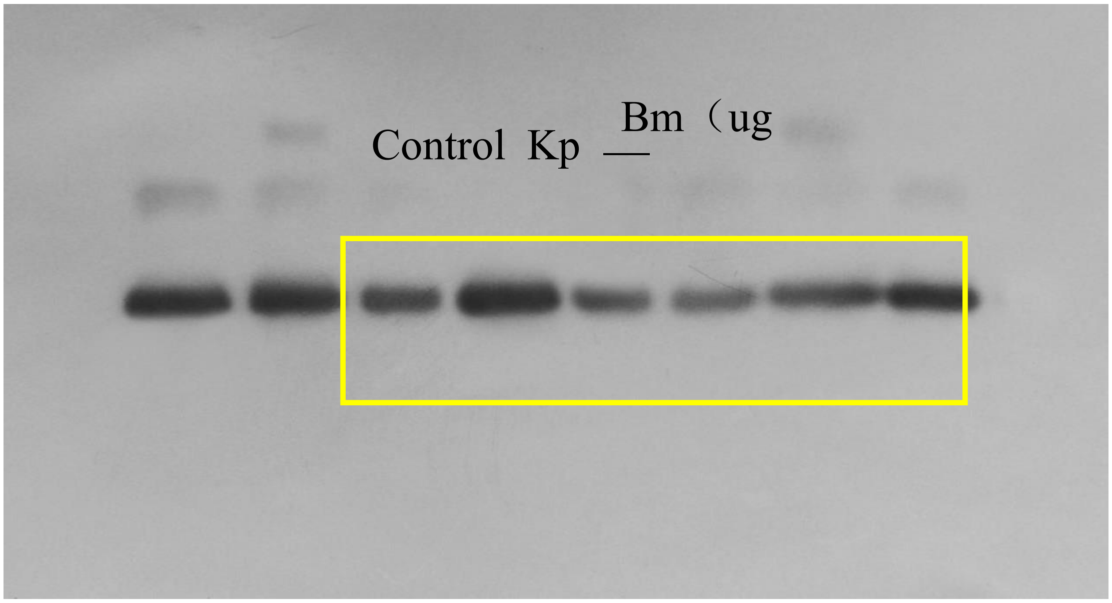

56497 52500 32760 61665 21787 23873 30694 35456

Figure 7. IkB

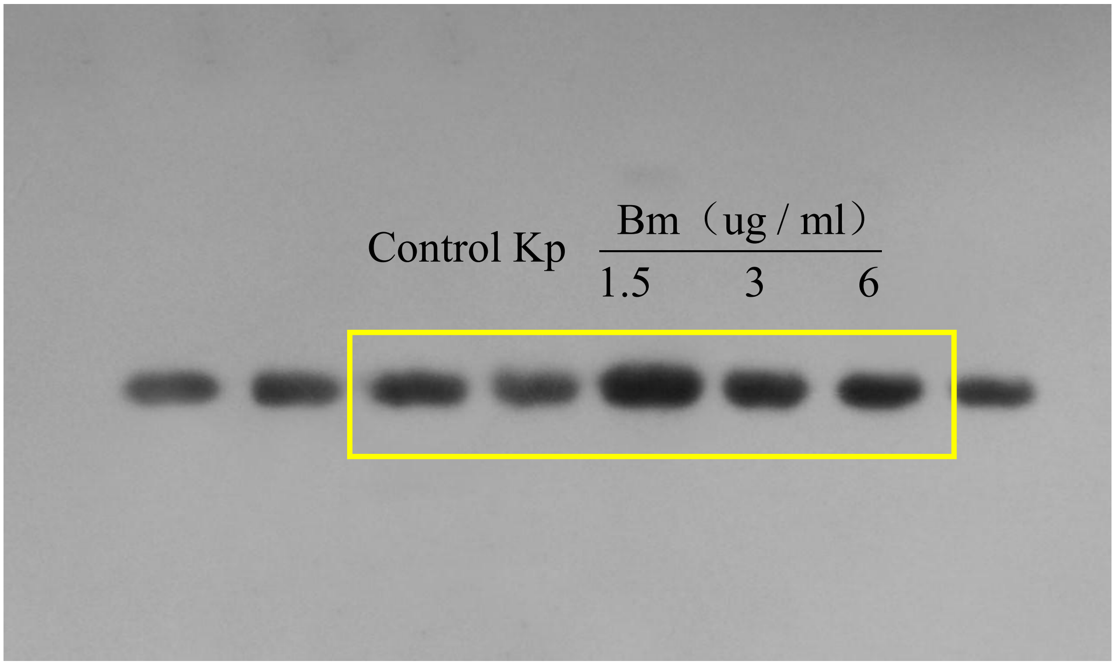

30910 34383 37869 16976 53455 34483 31979 22086

Figure 7. p-p65

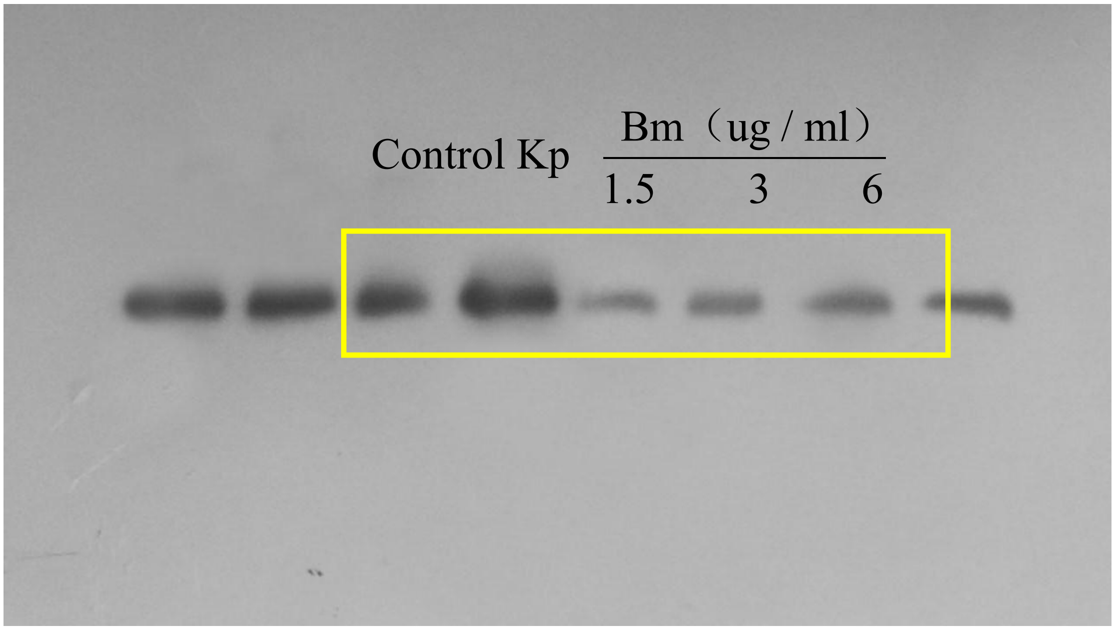

47457 44357 35839 53080 12542 15513 19891 22739

Figure 7. p65

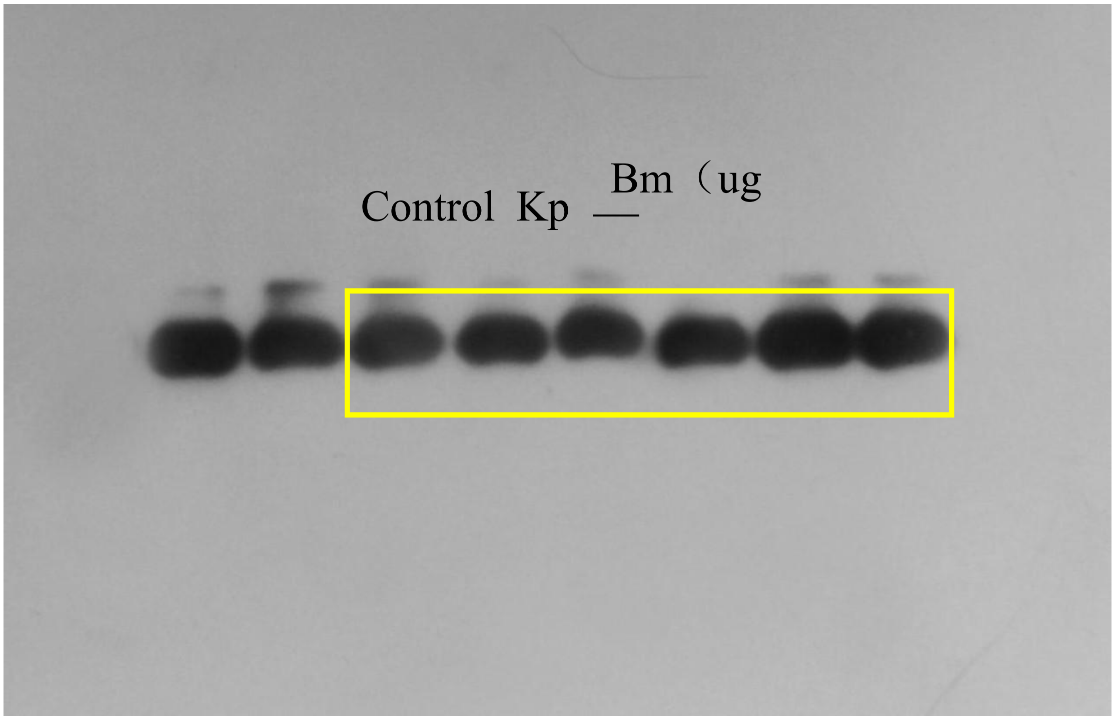

67980 67325 66691 64458 63716 65409 68124 66850

Figure 7. Actin

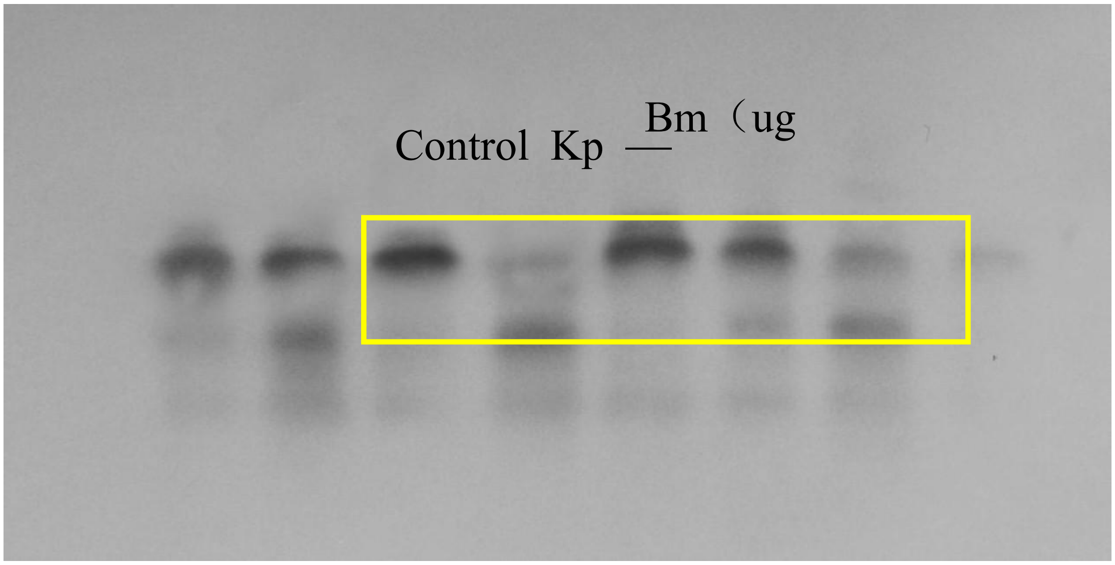

32292 39364 44290 7854 47735 34745 21612 8386

Figure 8. p-ERK

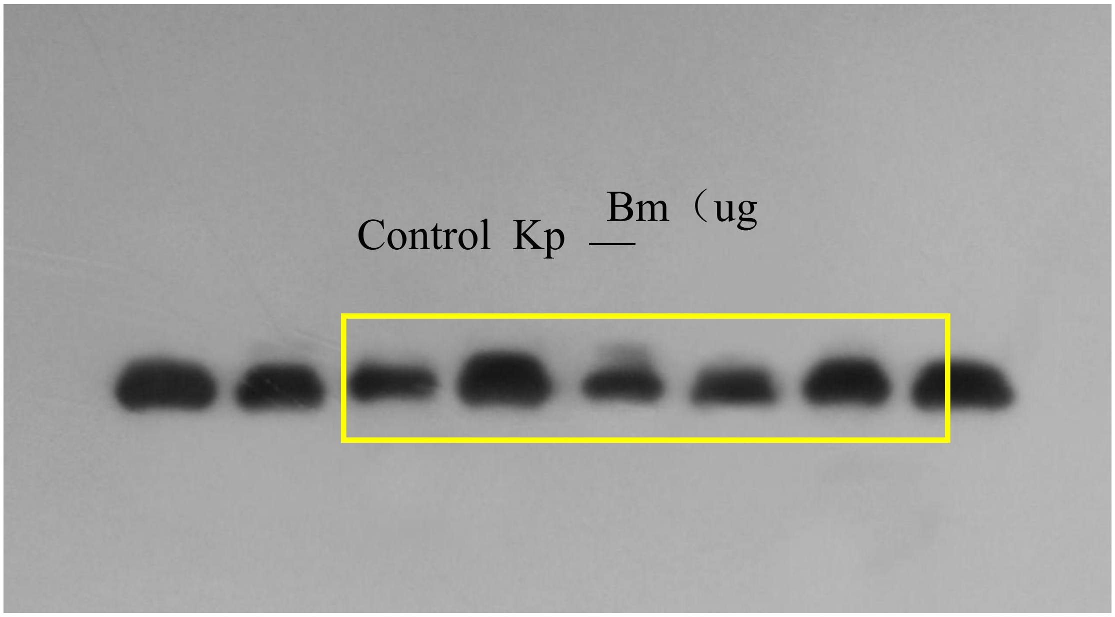

51977 40448 31095 53858 23479 28484 34519 40647

Figure 8. ERK

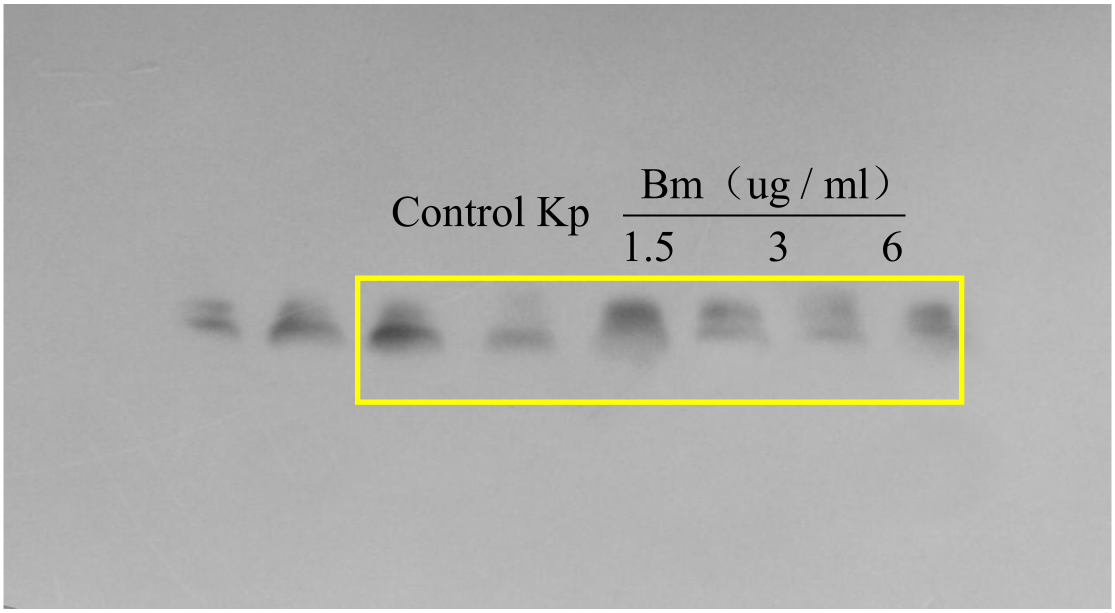

28002 35489 40737 18389 44405 30909 22081 20016

Figure 8. p-JNK

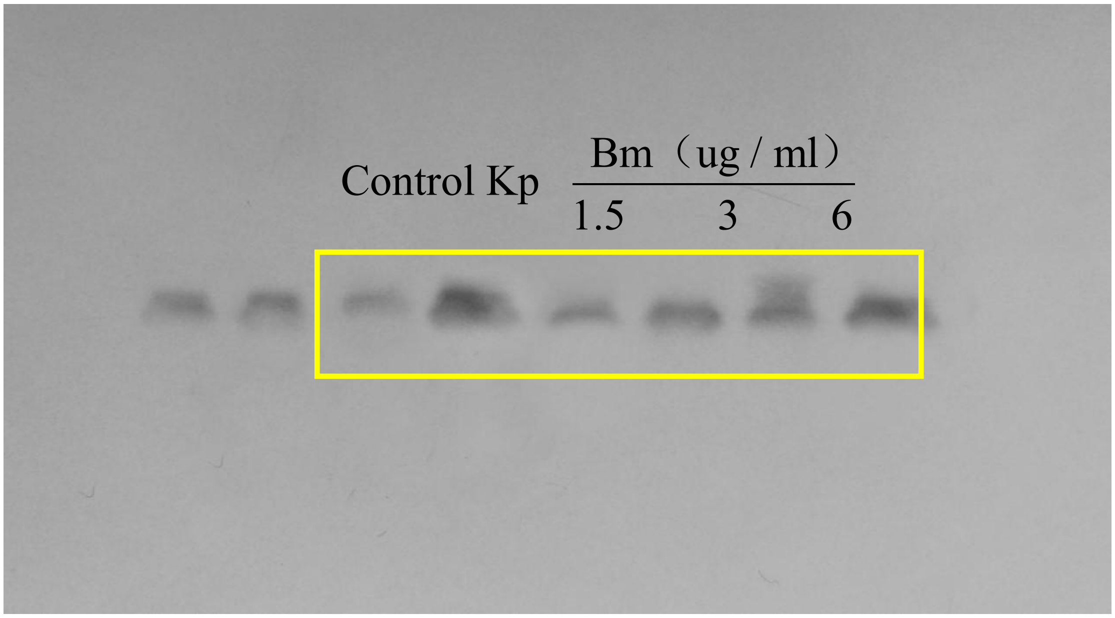

31309 26964 22944 43178 20611 28900 31422 34684

Figure 8. JNK

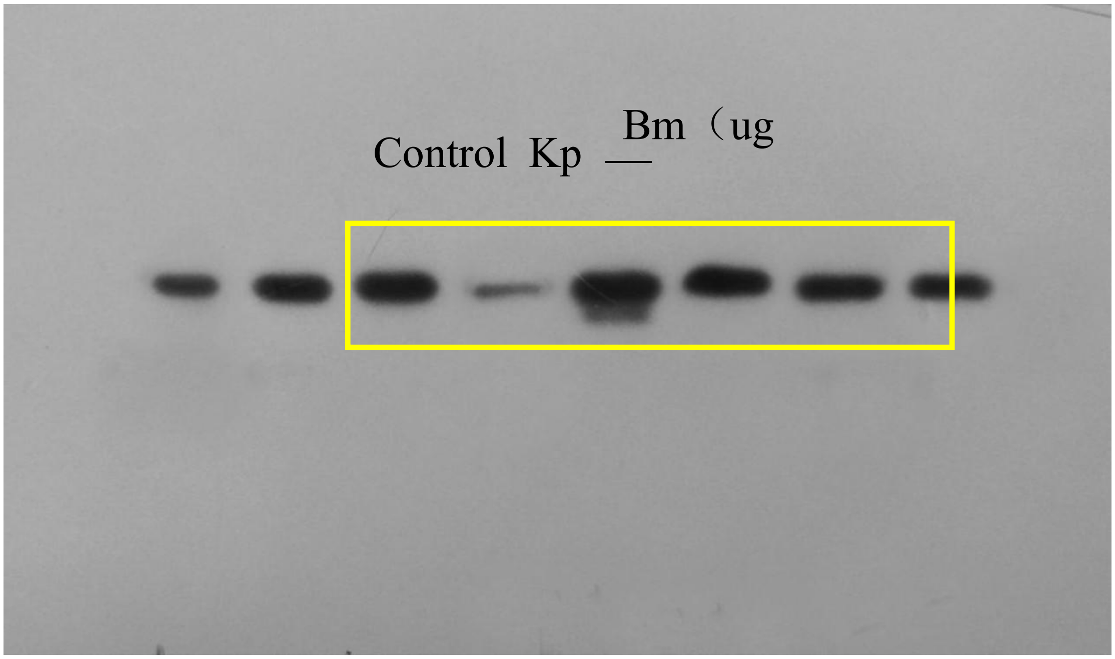

18918 25172 33473 14147 50747 39019 35386 27299

Figure7. P-P38

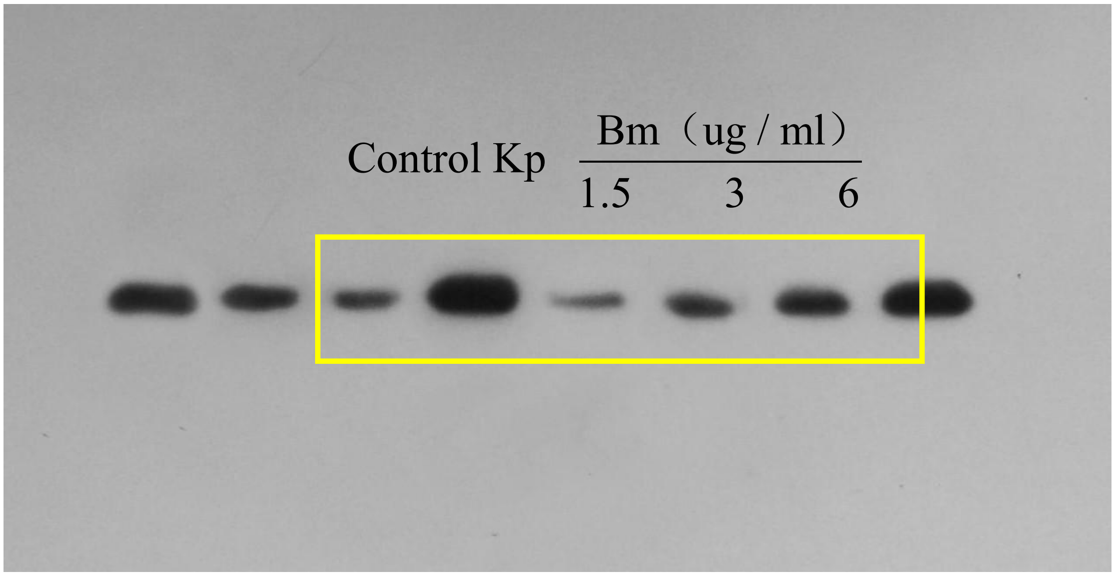

33850 28711 17622 51447 10469 19197 22100 36639

Figure7. p38

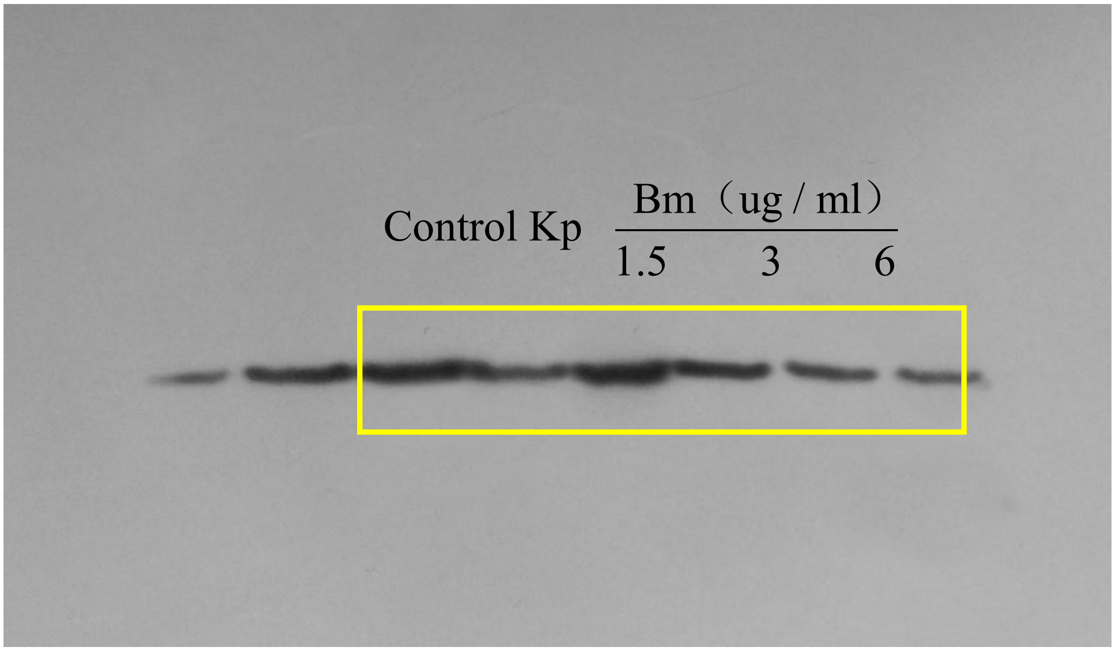

21243 44786 49663 14817 53201 35326 21954 17967

Figure 8. P-IkB

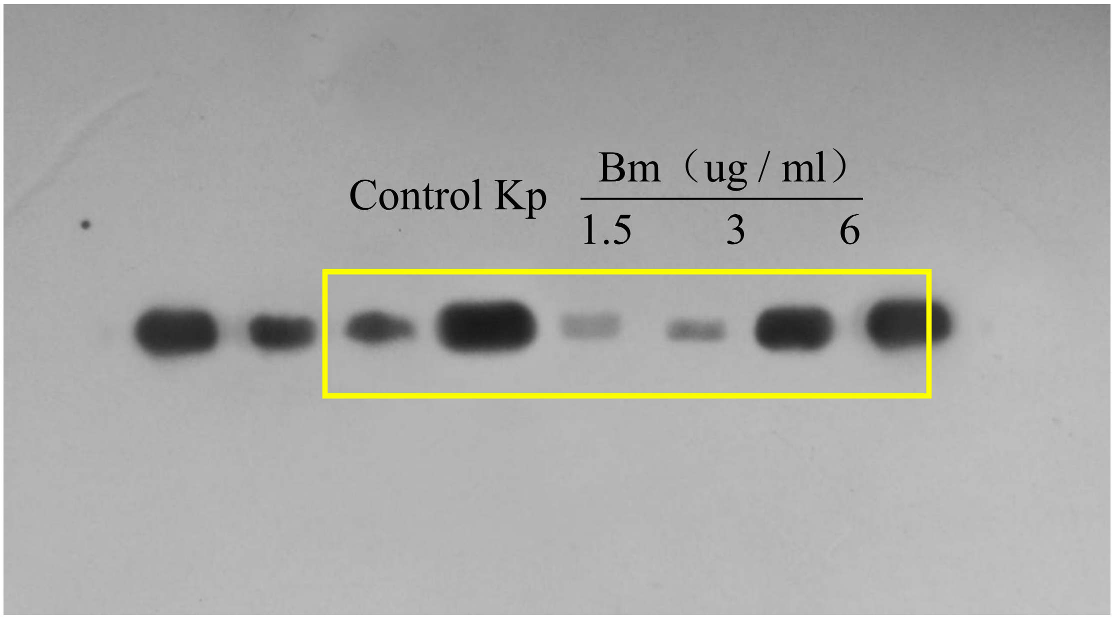

45990 31234 23128 54113 10584 12041 37294 45048

Figure7. IkB

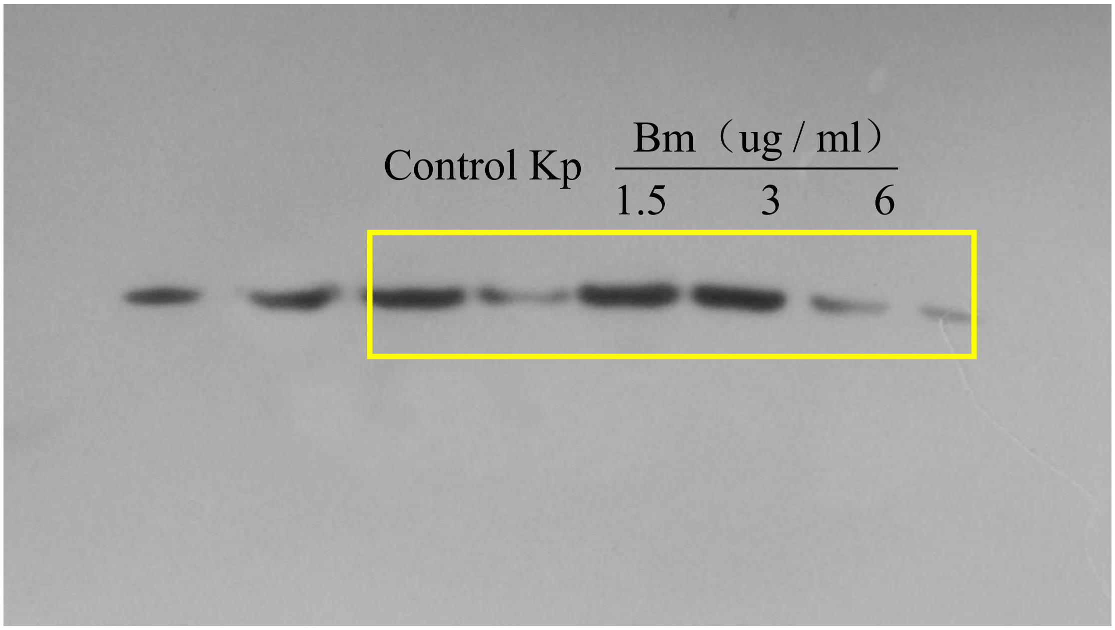

31100 38932 50862 13507 53782 49885 16734 6946

Figure7. P-P65

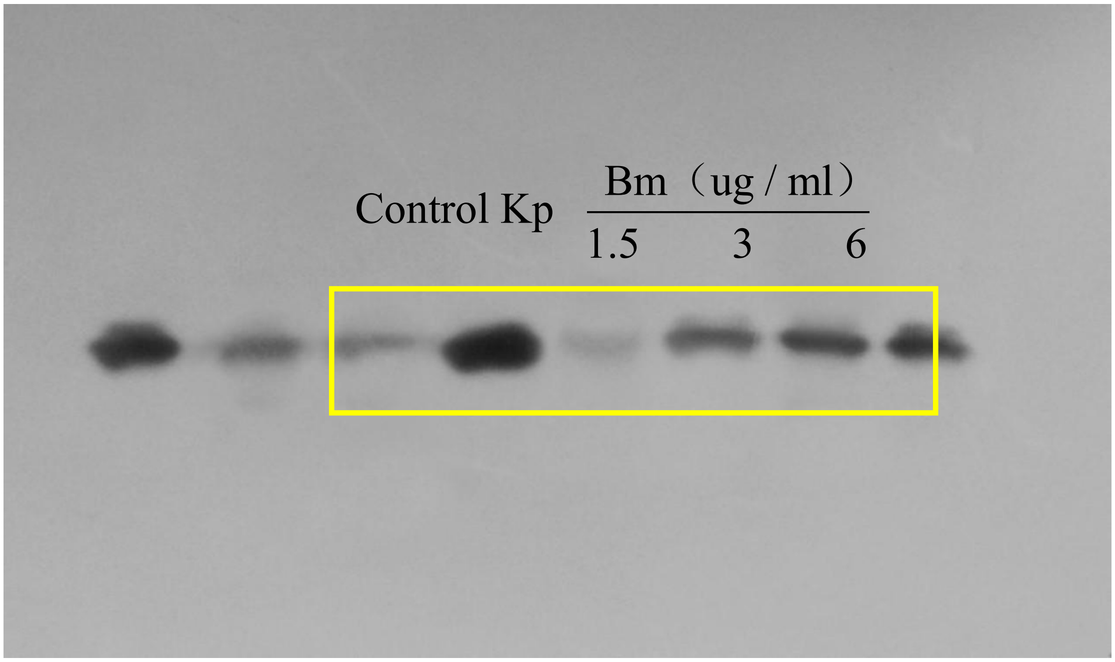

41170 26583 20082 49319 11191 20456 25620 29923

Figure 8. P65

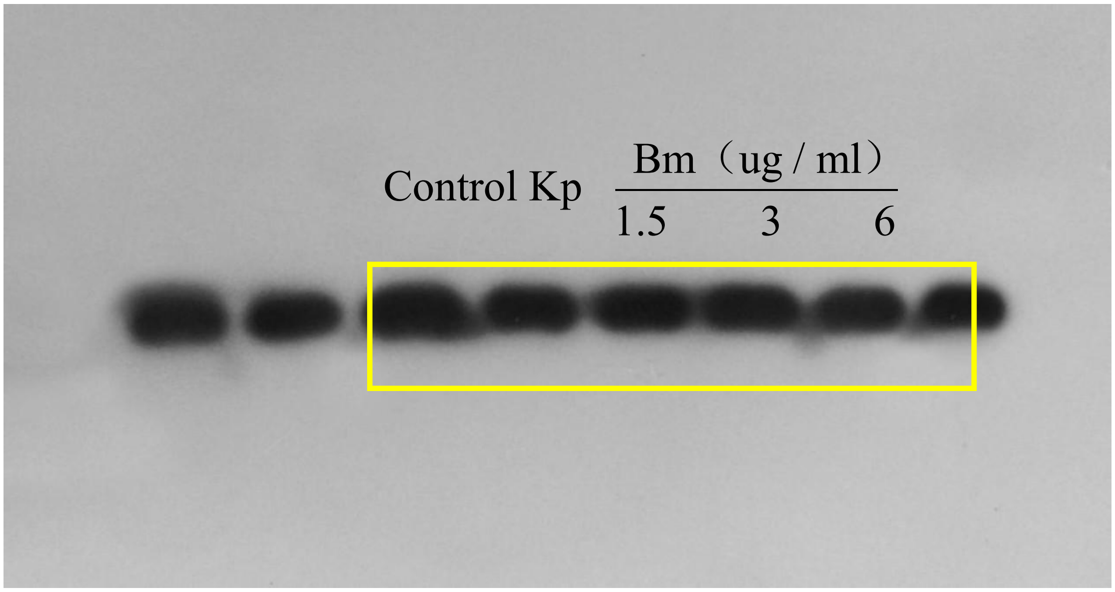

68942 67427 70257 66538 69321 68896 67812 65399

Figure 8. Actin
